# Absence of calcium-sensing receptor basal activity due to inter-subunit disulfide bridges

**DOI:** 10.1038/s42003-024-06189-3

**Published:** 2024-04-25

**Authors:** Shumin Ma, Xueliang Yin, Jean-Philippe Pin, Philippe Rondard, Ping Yi, Jianfeng Liu

**Affiliations:** 1 Cellular Signaling Laboratory, International Research Center for Sensory Biology and Technology of MOST, Key Laboratory of Molecular Biophysics of MOE, and College of Life Science and Technology, Huazhong University of Science and Technology, Wuhan, Hubei China; 2 Institut de Génomique Fonctionnelle (IGF), Université de Montpellier, CNRS, INSERM, Montpellier, Cedex 5 France

**Keywords:** Receptor pharmacology, Calcium and vitamin D

## Abstract

G protein-coupled receptors naturally oscillate between inactive and active states, often resulting in receptor constitutive activity with important physiological consequences. Among the class C G protein-coupled receptors that typically sense amino-acids and their derivatives, the calcium sensing receptor (CaSR) tightly controls blood calcium levels. Its constitutive activity has not yet been studied. Here, we demonstrate the importance of the inter-subunit disulfide bridges in maintaining the inactive state of CaSR, resulting in undetectable constitutive activity, unlike the other class C receptors. Deletion of these disulfide bridges results in strong constitutive activity that is abolished by mutations preventing amino acid binding. It shows that this inter-subunit disulfide link is necessary to limit the agonist effect of amino acids on CaSR. Furthermore, human genetic mutations deleting these bridges and associated with hypocalcemia result in elevated CaSR constitutive activity. These results highlight the physiological importance of fine tuning the constitutive activity of G protein-coupled receptors.

## Abstract (web summary)

G protein-coupled receptors oscillate between inactive and active states, resulting in constitutive activity. Here we show that the calcium-sensing receptor is strongly maintained in the inactive state, resulting in undetectable constitutive activity.

## Introduction

Cell surface receptors generate intracellular signals upon activation by external stimuli. Like all proteins, these receptors exhibit constant conformational dynamics oscillating between inactive and active states, which has been extensively studied for G protein-coupled receptors (GPCRs)^1–4^. Accordingly, receptors that maintain in an active state for sufficiently long periods can signal without activation by external stimuli^5–9^. Such constitutive activity can leads to an optimal physiological output under evolutionary pressure, which is biologically significant^10^. Indeed, many genetic mutations that have been found to either increase or decrease receptor constitutive activity can be the origin of human diseases^6,11,12^.

For most GPCRs, the constitutive activity can be easily detected^6,13–16^. However, some of them exhibit stable inactive conformations that lead to the undetectable constitutive activity, which permits receptors to generate signals only upon agonist activation. For instance, α_1B_ adrenergic receptor is well constrained in inactive state by a specific residue in the intracellular loop 3^17,18^. In addition, the inverse agonist cis-retinal stabilizes the rhodopsin in fully inactive state^19^. Conversely, some GPCRs exhibit strong constitutive activity that is almost equal to the activity elicited by agonist stimulation. As an example, the melanocortin-4 receptor, in which endogenous ligands (agouti-related proteins) act as inverse agonists^20^. The constitutive activity can be affected by the cellular environment. Indeed, constitutive activity of some metabotropic glutamate receptors (mGluRs), mGluR1 and mGluR5, is naturally inhibited by the long form of the intracellular partner Homer, but is revealed by the short form Homer1a^21^. Another example is the constitutive activity of 5-HT_6_ receptor, which is promoted by its interaction with neurofibromin^22^ or Cdk5^23^. GPCR constitutive activity can also be regulated by receptor phosphorylation^24^, dimerization^25^, alternative splicing^26^, or RNA editing^27^.

The discovery of inverse agonists and antagonists made it possible to identify the physiological significance of GPCR constitutive activity^6,10^. For example, Homer regulates the constitutive activity of mGluRs, which is important for synaptic metaplasticity^28,29^, and the constitutive activity of histamine H3 receptor plays a leading role in regulating the activity of histamine neurons^30^.

The class C GPCRs that sense amino-acids and their derivatives^31^ usually exhibit significant constitutive activity^21,32–34^. However, that of the calcium-sensing receptor (CaSR)^35^ has not been well studied. CaSR is a multifunctional receptor which plays important physiological role in organs involved or not involved in Ca^2+^ homeostasis^36,37^. The fundamental role of CaSR is to enable parathyroid gland to detect subtle changes in extracellular Ca^2+^ concentration and to respond by modulating the release of parathyroid hormone (PTH) in the opposite direction^38^. The result is Ca^2+^ normalization through the direct action of PTH, which promotes calcium release from bones and reabsorption by the kidney, in addition to its indirect effects in the gut. In the parathyroid gland, CaSR signaling activity suppresses PTH secretion, thus preventing the development of hypercalcemia. This role of CaSR is also mediated by cell-autonomous activities in kidney, bone and thyroid C cells. Consequently, if the receptor remains in a constant state of hyperactivity, it can lead to disturbances in calcium homeostasis and disorders such as hypocalcemia. Many genetic mutations that lead to loss- or gain-of-function of the CaSR have been identified in patients with metabolic syndromes, such as familial hypocalciuric hypercalcemia (FHH), neonatal severe primary hyperparathyroidism (NSPHT) or autosomal dominant hypocalcemia (ADH)^37,39^. In addition, CaSR autoantibodies that induced altered receptor signaling properties have been identified in acquired hypocalciuric hypercalcemia^40^. Actually, three commercial drugs can target CaSR as positive allosteric modulator (PAMs). Etelcalcetide^41,42^ targets the extracellular domain (ECD) and is applied in the treatment of secondary hyperparathyroidism, and two other drugs can bind to the transmembrane domain and are used to the treat both primary and secondary hyperparathyroidism^43,44^.

**Fig. 1:**
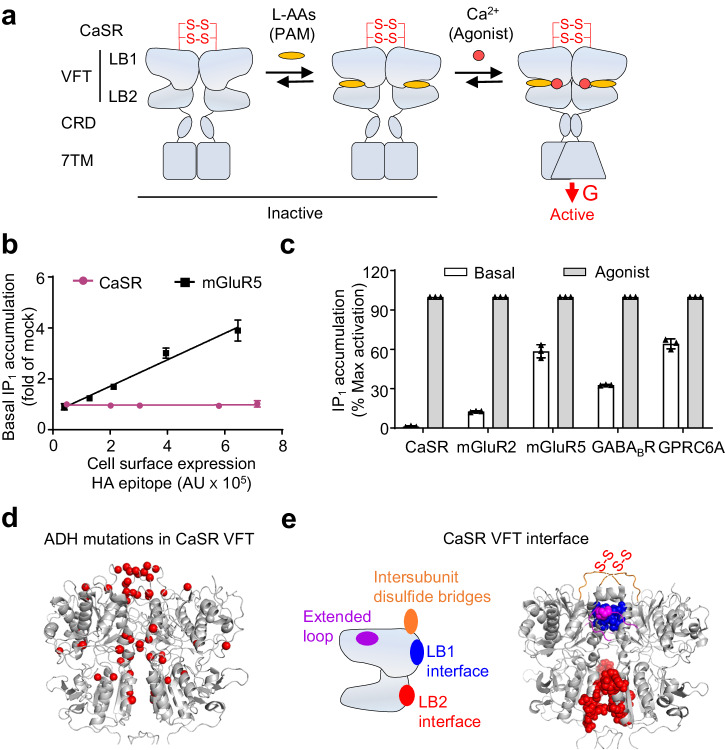
CaSR has very low constitutive activity. **a** Schematic diagram of CaSR activation mechanism. In CaSR, each subunit is composed of a VFT formed of two lobes, upper (LB1) and lower (LB2) lobe, that form Ca^2+^ and L-AAs binding site, a cysteine-rich domain (CRD) and a transmembrane domain (7TM) responsible for G protein activation. The two protomers are covalently linked through the two pair of inter-subunit disulfide bridges in the top of VFT. L-AAs act as pure-PAMs without direct agonist effect but could stabilizes the closed state of VFT. The active state can only be achieved by Ca^2+^ binding. **b** In both HA-tagged CaSR and mGluR5, basal inositol monophosphate (IP_1_) accumulation is proportional to the amount of receptors at the cell surface measured by ELISA. Data are mean ± SD from a typical experiment performed in triplicates (*n* = 3). **c** Basal IP_1_ accumulation for the indicated class C GPCRs. Data are mean ± SEM and normalized to the maximum response of each receptor (*n* = 3, 20 mM CaCl_2_ for CaSR, 1 mM glutamate for mGluR2 and mGluR5, 100 μM GABA for GABA_B_R and 100 mM L-Ala with 20 mM CaCl_2_ for GPRC6A). **d** Model showing location of autosomal dominant hypocalcemic (ADH) associated mutations in the CaSR VFT active structure (PDB: 7DTV). ADH mutations are highlighted as red spheres (α carbon). **e** Schematic and structure (PDB: 7M3E) showing the four main VFT dimer interface in CaSR.

Like other typical class C GPCRs, such as metabotropic glutamate receptors (mGluRs), γ-aminobutyric acid B receptor (GABA_B_R), GPRC6A and sweet/umami taste receptors, CaSR forms a constitutive dimer^45^. Each subunit within the dimer is composed of an extracellular Venus flytrap (VFT) domain, a cysteine-rich domain (CRD) and a seven-transmembrane (7TM) domain. Among them, the VFT domain is responsible for the binding of calcium ions^45^ and L-AAs^46^ (Fig. 1a). Furthermore, the two protomers are cross-linked by two pairs of disulfide bridges. Recently, the structure of full-length CaSR, both in inactive and active states with L-AAs, which shown to behave as pure-PAMs without direct agonist effect (Fig. 1a), or with allosteric modulators, including etelcalcetide, have been analyzed^47–54^. It remains unclear the role of L-AAs in the constitutive activity of CaSR.

Here, we demonstrate that in contrast to other class C GPCRs, CaSR displays no detectable constitutive activity. We reveal that the covalent disulfide bridges between the two subunits of this dimeric receptor are essential for the limitation of constitutive activity. More importantly, compare with the other class C GPCRs of the mGluR family that possess a single inter-subunit disulfide bridge, CaSR has two disulfide bridges. Both two disulfide bridges are essential for blocking the constitutive activity and potential agonist effect of ambient L-AAs. Several mutations of Cys residues that involved in these bridges had been identified in ADH patients. Interestingly, we found that all these mutations could generate constitutive activity, which revealed the importance of the disulfide bridges in controlling calcemia. These data further illustrate the significant role of the inter-subunit disulfide bridge in class C GPCRs in controlling their activity, and the importance of an evolutionarily optimized constitutive activity of receptors for proper physiological functions.

## Results

### Constitutive activity of CaSR is not detectable

We compared the constitutive activity of CaSR with mGluR5 by measuring the inositol phosphate-1 (IP_1_) accumulation, both of receptors are naturally coupled to the Gq protein. As reported previously, a high constitutive activity of mGluR5 could be detected^55^ (Fig. 1b and Supplementary Fig. 1a). In contrast, the IP_1_ constitutive accumulation in CaSR transfected HEK-293 cell was unable to be detected in the absence of calcium, even in the group with highest CaSR surface expression (Fig. 1b). Our data indicate that HEK-293 cells do not expressed endogenous CaSR at a level high enough to be detected (Supplementary Fig. 1b, c). The constitutive activity of mGluR2^32^, GABA_B_R^33,34^, and GPRC6A^56^ also can be detected in transfected cells as described in previous studies (Fig. 1c and Supplementary Fig. 1a). Co-transfected chimeric Gqi_9_ made it possible to detect the IP_1_ accumulation in HEK-293 cells that transfected with mGluR2 and GABA_B_R, for both receptors belong to the Gi/o coupled-receptors^57^. Altogether, these data showed that the CaSR activity is tightly controlled with a no detectable constitutive activity toward the Gq pathway.

### Undetectable constitutive activity of CaSR is due to the inter-subunit disulfide bridges

To identify the molecular basis for the undetectable constitutive activity in CaSR, we analyzed the numerous missense genetic mutations that have been reported to increase the activity of CaSR in patients with ADH disease^39^. Many mutations occurred in the VFT domain (Fig. 1d), especially at the dimer interface that is known to control the structural dynamics and receptor activation^2,4,47,58–60^. Based on the inactive and active structures of CaSR, there was reasonable to explain that the VFT dimer interface mainly consists of four regions^48,51,58^ (Fig. 1e). The upper loop contains a pair of cysteine residues (Cys129 and Cys131) which were responsible for the covalent link between the two protomers, and was highly conserved in CaSR during evolution (Supplementary Fig. 2a). Interestingly, this upper loop contains a hot spot of ADH mutations^39^.

**Fig. 2:**
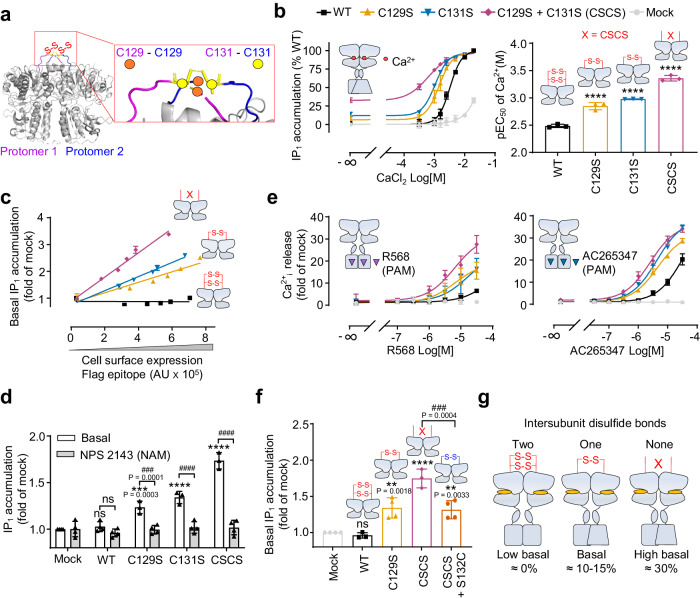
The inter-subunit disulfide bonds block the constitutive activity of CaSR. **a** Close-up view of the upper loop where the two cysteine residues Cys129 (orange circle) and Cys131 (yellow circle) critical for the formation of two inter-subunit disulfide bonds (PDB: 7M3E). Of note, in this structure, only the two Cys131 forms a disulfide bridge are shown, meanwhile in another structure (PDB: 7DTV) this is the two Cys129 that forms a disulfide bridge. This variability between CaSR structures is most probably due to the high flexibility of this upper loop. **b** IP_1_ accumulation induced by CaCl_2_ in HEK-293 cells transfected with the CaSR WT or indicated mutants and the corresponding potencies (*n* = 3, pEC_50_ = −logEC_50_). **c** In both Flag-tagged WT receptor and indicated mutants, basal IP_1_ accumulation is proportional to the amount of receptors at the cell surface measured by ELISA. Data are mean ± SD from a typical experiment performed in triplicates (*n* = 3). **d** Effect of NAM NPS-2143 (10 μM, pretreated for 1.5 h) on the basal IP_1_ accumulation measured for the WT and indicated mutants (*n* = 4). **e** Intracellular calcium release for the WT and indicated mutants stimulated by PAM R568 (*n* = 5) or ago-PAM AC265347 (*n* = 4) in the absence of ligands. **f** Basal IP_1_ accumulation measured for the WT and indicated mutants (*n* = 4). **g** Schemes illustrating the link between the number of inter-subunit disulfide bonds and the basal activity of CaSR. Data above are mean ± SEM of at least three biologically independent experiments each performed in triplicates and normalized to mock (**c**, **d**, **e**, **f**) or the WT (**b**). Significance was analyzed using one-way ANOVA with Dunnett’s multiple comparisons (**b**, **f**) or two-way ANOVA with Sidak’s multiple comparisons (**d**) with ****P ≤ 0.0001, **P ≤ 0.01, ns for *P* > 0.05 versus the mock (**d**, **f**) or the WT (**b**), and ^####^*P* ≤ 0.0001, ^###^*P* ≤ 0.001, ns for *P* > 0.05 compared with indicated groups.

We therefore investigated the role of the inter-subunit disulfide bridges between the two upper loops (Fig. 2a). These Cys were replaced with Ser since such natural mutations have been previously reported in ADH^61,62^. We found that C129S + C131S (CSCS) double mutations resulted in the appearance of monomer band in non-reducing SDS-PAGE experiments, however, dimeric form of the mutant CSCS still could be detected on the cell membrane (Supplementary Fig. 2b, c). Indeed, dimers and even larger oligomers are observed for many GPCRs in SDS-PAGE even when they are not covalently linked (i.e. many class A GPCRs). This is likely due to the consequence of unfolding by SDS and mixing the two chains during this process, resulting in non-covalent dimers that are not sensitive to reducing agents such as DTT^63^. We further verified this result by bioluminescence resonance energy transfer (BRET) and obtained a similar conclusion, which suggested that the dimerization of CaSR was maintained by multiple interactions (Supplementary Fig. 2d). Interestingly, both single and double mutants displayed strong constitutive activity, despite their surface expression was similar to wild-type (WT) receptor (Fig. 2b and Supplementary Fig. 2e). The constitutive activity was positively correlated with the cell surface expression of the mutated receptors (Fig. 2c) and could be blocked by the CaSR negative allosteric modulator NPS 2143^64^ (Fig. 2d). The mutants also showed a significant increase in CaCl_2_ potency compared with WT receptor (Fig. 2b). In addition, in contrast to the WT receptor, without agonist binding, the mutants became more sensitive in the activation induced by PAMs, NPS R568 or AC265347, both of which bind to the 7TM domain of CaSR^65,66^ (Fig. 2e). Similar results were obtained by measuring the phosphorylated ERK1/2 (pERK), these Cys mutants upregulated the basal level of pERK and NPS-2143 treatment inhibited this process (Supplementary Fig. 3a). Altogether, these results showed the absence of inter-subunit disulfide bonds led to a high basal activity of CaSR.

To further validate the correlation between inter-subunit disulfide bond and basal activity, we have re-built one inter-subunit disulfide bond by changing the Ser132 into Cys in the CSCS construct. This mutant showed a similar dimerization pattern as the WT and single Cys mutants in non-reducing SDS-PAGE experiments (Supplementary Fig. 3b). Interestingly, the CSCS + S132C construct showed a strong reduction of constitutive activity in both IP_1_ accumulation (Fig. 2f) and ERK phosphorylation when compared with the CSCS construct (Supplementary Fig. 3c). Furthermore, addition of this S132C mutation in the CSCS construct could restore the Ca^2+^ pEC_50_ similar to the single Cys mutant (Supplementary Fig. 3d). Of note, an another CaSR construct where both Cys129 and Cys131 were replaced by Ala also showed a similar increase in basal activity, that could be inhibited by NPS2143 (Supplementary Fig. 3e). These results further indicated that the emergence of the constitutive activity in CaSR is a result of inter-subunit disulfide bridges removal. Taken together, our data suggested that the inter-subunit disulfide bridges maintain the inactive state of CaSR and were required to restricting the basal activity of receptor. Interestingly, our data also revealed that the presence of two disulfide bridges were crucial to the strong limitation of the basal activity, since a single mutation of Cys129 or Cys131 led to a moderate constitutive activity (10–15% of the WT Emax; see Fig. 2b). And the double mutations that totally remove inter-subunit covalent link between VFTs generated a high constitutive activity (30% of the WT Emax) (Fig. 2g).

### The inter-subunit disulfide bridge favors the basal activity of mGluRs

**Fig. 3:**
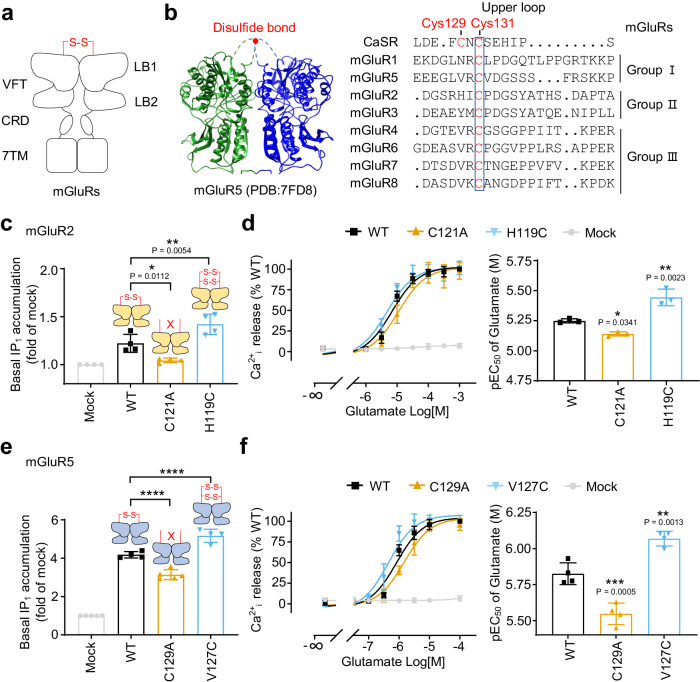
The inter-subunit disulfide bridge favors the basal activity of mGluRs. **a** Schematic representation of the mGluR homodimers. **b** Structure of mGluR5 VFT (PBD: 7FD8) and the sequence alignment of the upper loop of the human CaSR (Cys 129 and Cys 131 are indicated) and rat mGluRs using Clustal Omega and ESPript 3. CaSR is used as reference for residue numbering and the blue box indicated the most conserved residues. **c**, **e** Basal IP_1_ accumulation for the WT and indicated mutants of mGluR2 (**c**, *n* = 4) of mGluR5 (**e**, *n* = 5). **d**, **f** Intracellular calcium release mediated by the indicated mutants of mGluR2 (**d**, *n* = 6) or mGluR5 (**f**, *n* = 5) upon stimulation with glutamate and the corresponding pEC_50_. Data above are mean ± SEM for each individual experiment and normalized to the mock (**c**, **e**) or the maximum response of WT (**d**, **f**). Significance was analyzed using one-way ANOVA with Dunnett’s multiple comparisons with ****P ≤ 0.0001, ***P ≤ 0.001, **P ≤ 0.01, and *P ≤ 0.05 versus the WT.

Since the inter-subunit disulfide bridge is conserved in most class C homodimers, especially in mGluRs (Fig. 3a), we wondered whether they play a similar role in controlling the basal activation. In contrast to CaSR, mGluRs have only one conserved Cys residue in the upper loop that could establish single disulfide bridge (Fig. 3b). It was reported that mutated Cys121 to Ala in mGluR2 stabilized the VFT in inactive state^4^. Consistently, we found that the C121A mutant has a lower constitutive activity in IP_1_ accumulation than the WT receptor when co-transfected with Gqi9 (Fig. 3c) and this decrease was not due to the difference in cell surface expression (Supplementary Fig. 4a). In addition, to mimic the upper loop of CaSR and potentially favor the formation of two inter-subunit disulfide bridges, a second Cys residue was introduced at the position 119 in mGluR2 equivalent to the first conserved Cys (Cys129) in the CaSR (Fig. 3c and Supplementary Fig. 4a, b). The H119C mutation significantly increased the basal activity of mGluR2 (Fig. 3c) as well as its potency for glutamate (Fig. 3d). Similar experiments were also performed on mGluR5, which belongs to another group of mGluRs that are naturally coupled to the Gq—phospholipase C signaling pathways. Similar to the mGluR2 mutants, deletion the inter-subunit disulfide bridge (C129A) in mGluR5 resulted in a lower basal activity, while an additional Cys (V127C) could increase the basal activity (Fig. 3e and Supplementary Fig. 4c, d) and its potency for glutamate (Fig. 3f). Altogether, our results showed that the inter-subunit disulfide bridge controls the basal activity of CaSR and mGluRs in distinct ways.

### Removal of inter-subunit disulfide bridge enhances signaling output of CaSR

CaSR could respond to diverse ligands and signals through different G proteins^67,68^. We investigated whether the CSCS mutant responds differently to these ligands and show preference toward different G proteins. As mentioned above, the CSCS mutant was more sensitive to Ca^2+^ in comparison with WT CaSR, and the results from other ligands like Mg^2+^, neomycin and spermine show a similar effect of these ligands (Supplementary Fig. 5a). To investigate the influence of this mutant on the activation of different Gq, G_i/o_ and G_13_ proteins, we measured the G protein dissociation by using BRET-based sensors as previously described (Supplementary Fig. 5b)^34,69^. For all these G proteins, the BRET signal between the Gα and Gβγ subunits was lower in the CSCS group than WT under basal conditions (Supplementary Fig. 5c–i). Upon agonist stimulation, the BRET signal measured for most G proteins showed increased pEC50 and decreased Emax except G_13_, which behaved differently (Supplementary Fig. 5c–j). These results demonstrated that removal of the inter-subunit disulfide bridges in CaSR did not influence G protein selectivity under basal and agonist-induced conditions.

### Genetic mutations at Cys129 and Cys131 favor the constitutive activity of CaSR

**Fig. 4:**
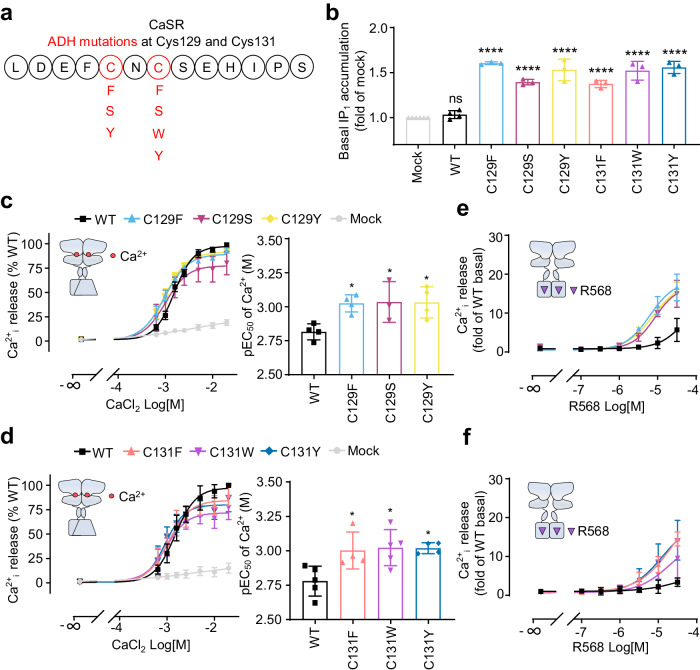
Genetic mutations at Cys129 and Cys131 favor the constitutive activity of CaSR. **a** Scheme showing the reported ADH mutations at Cys129 and Cys131. **b** Basal IP_1_ accumulation measured for the WT and indicated mutants (*n* = 3). **c**, **d** Intracellular calcium release in WT and indicated mutants stimulated with CaCl_2_ and the corresponding pEC_50_ (*n* = 3–5). **e**, **f** Intracellular calcium release measured for the WT and indicated mutants stimulated by PAM R568 in the absence of ligands (*n* = 3–5). Data above are mean ± SEM of at least three independent experiments performed in triplicates and normalized to the mock (**b**) or the WT (**c**–**f**). Significance was analyzed using one-way ANOVA with Dunnett’s multiple comparisons with ****P ≤ 0.0001, **P ≤ 0.01, *P ≤ 0.05, and ns for *P* > 0.05 versus the mock (**b**) or the WT (**c**, **d**).

Among the genetic gain-of-function mutations that are associated with ADH (Fig. 1d), several mutations occurred at Cys129 or Cys131 (Fig. 4a)^61,70^. We tested the effect of six natural single mutations on the basal activity of CaSR and found that all these mutants showed a strong basal activity (Fig. 4b) despite their surface expression was similar to WT receptor (Supplementary Fig. 6a). Furthermore, compared with WT receptor, these mutants showed higher Ca^2+^ potency and more sensitive to NRS R568 (Fig. 4c–f). In addition, using the BRET-based G protein sensors, we showed that the mutant C131W, used as an example, has a higher basal activity in different G protein pathways than WT receptor, and an increased pEC_50_ and decreased E_max_ in C131W also been revealed which were in consistence with the results obtained with the CSCS construct. We also found that the CSCS construct showed higher sensitivity to CaCl_2_ compared to this C131W mutant except for the G_oB_ pathway (Supplementary Fig. 6b–j). Therefore, the natural mutations at either C129 or C131 would impair the integrity of the inter-subunit disulfide bridges and led to the basal activation of CaSR.

### L-AAs binding in CaSR is required for the basal activity of the CSCS construct

**Fig. 5:**
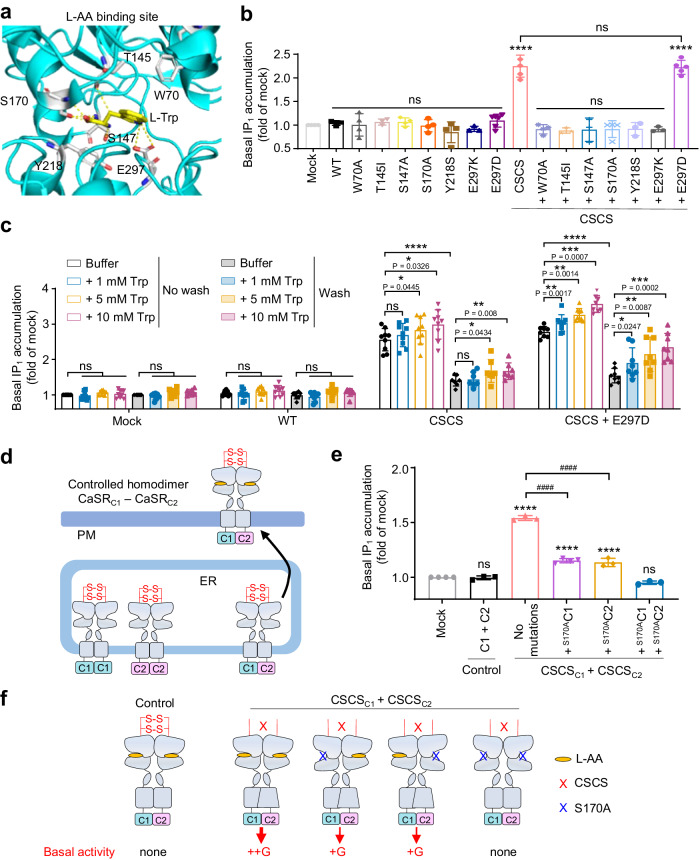
L-AA binding is required for the basal activity of CSCS. **a** View of the six residues involving in the L-Trp binding in the CaSR structure (PDB: 7DTW). **b** Basal IP_1_ accumulation measured for the WT and indicated mutants (n = 5). **c** Basal IP_1_ accumulation for the WT and the indicated mutants in the presence of increasing concentrations of Trp under conditions without or with three wash steps over a 3-h period (*n* = 9). **d** Cartoons illustrating the mechanism of only the homodimer formed by the CaSR_C1_ and CaSR_C2_ subunits could reach the cell surface. **e** Basal IP_1_ accumulation mediated by the indicated subunit compositions (*n* = 4). **f** Schemes illustrating how the mutations of the glutamate binding pocket (S170A) in the CaSR_C1-C2_ homodimer impair the basal activity of the receptor. Data above are mean ± SEM of at least three independent experiments performed in triplicates and normalized to the mock. Significance was analyzed using one-way ANOVA with Dunnett’s multiple comparisons with ****P ≤ 0.0001, ***P ≤ 0.001, **P ≤ 0.01, *P ≤ 0.05 and ns for *P* > 0.05 compared with indicated groups, and ^####^*P* ≤ 0.0001 versus the indicated CSCS construct containing no additional mutations.

It was recently reported that ambient L-AAs in the cell culture medium were constitutively bound in the CaSR VFT (Fig. 5a), and act as pure-PAMs during activation by extracellular Ca^2+^ ^45,47–53^. However, the extracellular concentrations of these L-AAs in cellular assays were difficult to control^32,45^, even in the absence of serum. To study the importance of the L-AAs in the basal activity of the CSCS mutant, we constructed several single mutations that are known to prevent L-AAs binding in the VFT binding pocket of CaSR^45,47,49^. Interestingly, the mutations abolished the constitutive activity of CSCS mutant (Fig. 5b and Supplementary Fig. 7a). Of note, the gain-of-function mutation E297D^71^ that was proposed to increase the affinity of L-AAs for CaSR^45^ had no effect on the basal activity of CSCS (Fig. 5b). Then we washed the cells three times with starvation buffer over 3 h to remove the L-AAs that had bound to the receptor, and discovered that the basal activity of the mutations were significantly decreased in this group, and it could been recovered by the addition of L-Trp (Fig. 5c). Elution and rebinding treatments with L-AAs showed that the spontaneous activity of the CSCS mutant was dependent on the intact amino acid binding. Moreover, this agonistic effect of L-AAs was more significant with the CSCS + E297D construct, which is consistent with the higher sensitivity of the CSCS + E297D construct to L-AAs conferred by the mutation E297D^45^. The results suggested that removal of the inter-subunit disulfide bridges altered the pure-PAM effect of L-AAs that become ago-PAMs.

We further investigated whether the binding of ambient L-AAs to both VFTs of the dimer was required to maintain the basal activity. We constructed a ‘controlled’ CaSR homodimer formed by the CaSR_C1_ and CaSR_C2_ subunits^51,72–74^. In these constructs, the C-terminus of the CaSR subunits were replaced by the C-terminus from modified GABA_B1_ (C1) or GABA_B2_ (C2) subunits, respectively. The dimers that consisted of the same C-terminus (CaSR_C1_ + CaSR_C1_ or CaSR_C2_ + CaSR_C2_) hardly reached the cell surface (Fig. 5d and Supplementary Fig. 7b) and responded weakly to the CaCl_2_ stimulation (Supplementary Fig. 7c). Then we introduced a S170A single mutation to the CSCS_C1_-CSCS_C2_ constructs. This mutation that occurs in CSCS_C1_ or CSCS_C2_ alone strongly decreased the basal activity, and more importantly, when S170A mutation is in both VFTs it abolished the constitutive activity (Fig. 5e and Supplementary Fig. 7d). These results suggested that L-AA binding in at least one VFT of CSCS was required to generate constitutive activity, and binding in both VFTs could produce the strongest basal activity (Fig. 5f). Altogether, these data showed the importance of L-AAs induced VFT closure in the basal activity of CSCS.

### Upper interface of the VFT is not involved in CaSR basal activity

In addition to the inter-subunit disulfide bridges, we wondered whether other regions at the VFT interface could control the constitutive activity of CaSR. Other direct interactions between the two LB1 were the extended loops which could stabilized the VFT interface through hydrophobic and polar interactions (Fig. 1e and Supplementary Fig. 8a). This unique structure is not conserved in the other class C GPCRs (Supplementary Fig. 8b). Two loss-of-function genetic mutations in this loop, S53P and P55L^39,70^ (Supplementary Fig 8a) showed no effect on the basal activity of the receptor (Supplementary Fig. 8c, d). But both mutants have a lower potency for calcium activation (Supplementary Fig. 8e), which were in line with their classification as inactivating mutations. In addition, we analyzed another region of the LB1 interface that is conserved in the other class C GPCRs (Supplementary Fig. 8f, g). The two loss-of-function genetic mutations in this region, L159F and L159R^75,76^, as well as L108A, L112A, and L156A had no effect on the basal activity of receptor (Supplementary Fig. 8h, i), but decreased the pEC_50_ of Ca^2+^ (Supplementary Fig. 8j). On the other hand, the residue Phe160 located immediately after helix C, which may interact directly with the upper loop (residue Val115) in both the inactive and active states (Supplementary Fig. 8k), was also mutated. Interestingly, the F160A mutant shows a significant constitutive activity (Supplementary Fig. 8l) and higher Ca^2+^ potency than the WT (Supplementary Fig. 8j). This is probably due to an effect on the conformation of the upper loop via the loss of interaction with the side chain of Val115, which could lead to its repositioning. Indeed, Val115 is located between the end of helix B and the beginning of the upper loop that adopts a helical conformation, a critical position for the relative positioning of helix B and the upper loop (Supplementary Fig. 8k). Altogether, our data showed that the LB1 interface of the VFTs, apart from the upper loop containing the disulfide bridges, is not involved in CaSR basal activity.

### Negative charges in the lower interface of the VFT limit CaSR activation but is not involved in basal activity

**Fig. 6:**
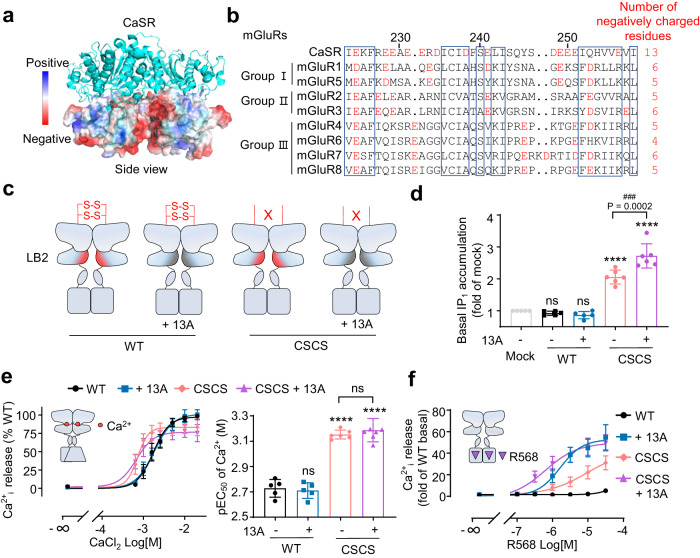
Negative charges in the lower interface of the VFT limit CaSR activation but is not involved in basal activity. **a** Electrostatic potential map showing charged residues in CaSR LB2 interface in the active state (PDB: 5FDK). **b** Sequence alignment of the LB2 interface of the human CaSR and rat mGluRs using Clustal Omega and ESPript 3. CaSR is used as reference for residue numbering and the blue boxes indicated the conserved residues. **c** Scheme showing constructs of where the negatively charged residues of LB2 interface were mutated into alanine (13 A) in the background of the WT and CSCS. 13 A includes mutations at residues E224, E228, E229, E231, E232, D234, D238, E241, D248, E249, E250, E251, and E257. **d** Basal IP_1_ accumulation for the CaSR the WT and indicated mutants (*n* = 6). **e** Intracellular calcium release induced by CaCl_2_ in the WT and indicated mutants and the corresponding pEC_50_ (*n* = 7). **f** Intracellular calcium release measured for the WT and indicated mutants stimulated by PAM R568 in the absence of ligands (*n* = 4). Data above are mean ± SEM of at least four independent experiments performed in triplicates and normalized to the mock (**d**) or the WT (**e**, **f**). Significance was analyzed using one-way ANOVA with Dunnett’s multiple comparisons with ****P ≤ 0.0001 and ns for *P* > 0.05 versus the mock (**d**) or the WT (**e**), and ^###^*P* ≤ 0.001 versus the CSCS.

In contrast to mGluRs and other class C GPCRs, the lower interface of the CaSR VFT (LB2) was highly enriched with negatively charged residues (Fig. 6a, b). Our recent study have ruled out the possibility that this region has a functional binding site for Ca^2+^ ^45^. To investigate the effect of LB2 interface on basal activity, we constructed a mutant where all the 13 negatively charged residues in the acidic patch been replaced by alanine, and we named this mutant 13 A (Fig. 6c, Supplementary Fig. 9a). The 13 A mutant showed no effect on basal activity when made in the WT receptor, while the introduction of 13 A mutation in the CSCS construct further increased the basal activity (Fig. 6d). In addition, the similar pEC_50_ for Ca^2+^ indicated that introduction of 13 A mutations did not impair the activation of CaSR by Ca^2+^, both in WT and CSCS constructs (Fig. 6e), which was consistent to the results obtained with single or multiple mutants in this acidic LB2 patch in previous study^45^. Interestingly, the 13 A mutations potentiated the activation of CaSR by NPS R568, both in WT and CSCS constructs (Fig. 6f). Finally, we analyzed the effect of the PAM etelcalcetide, which could form a disulfide bond with the free LB2 Cys482^42^ and binds to the LB2 interface by forming salt bridges with the patch of negatively charged residues^51^ (Supplementary Fig. 9b). Etelcalcetide showed no impact on the basal level of IP_1_ accumulation in WT receptor, but elevated the basal activity of the CSCS construct (Supplementary Fig. 9c). Moreover, etelcalcetide could increase the Ca^2+^ potency of WT and CSCS mutant (Supplementary Fig. 9d), as well as the agonist effect of NPS R568 on both WT and CSCS constructs (Supplementary Fig. 9e). Altogether, our results showed that the negatively charged interface of LB2 exerted no effect on constitutive activity of WT receptor but could enhance it in the CSCS construct, underscoring the pivotal role of inter-subunit disulfide bonds in limiting the constitutive activity of CaSR. It also suggests that when both LB2s could come closed, it facilitates the activation of CaSR by PAM binding in the 7TM.

## Discussion

As a special nutrient-sensing receptor regulated by various ligands, CaSR develops a unique ability to sense the tiny fluctuation of extracellular Ca^2+^ ^77,78^. This response is essential for tight control of calcemia through various mechanisms, including in the parathyroid which controls PTH release, in the bones and kidney among others^37^. Therefore, the CaSR activity was critical for proper physiological response. In this study, we showed that inter-subunit disulfide bridges functions as molecular locks to significantly restrict the constitutive activity. They also reduce receptor sensitivity to the agonists, as well as inhibit the agonism of CaSR by natural and synthetic PAMs, thus setting the Ca^2+^ potency to an optimal value.

Class C GPCRs are complex GPCRs^79^, the majority of them have constitutive activity^34,55,79–81^. Their activation mechanism involves several steps, such as the closure and reorientation of VFT, which leads the CRD closed to each other, followed by a direct interaction between the 7TM domains through TM6 and then a conformational change in one of the 7TM domain^60^. Consequently, their constitutive activity can originate from various structural elements. First, as demonstrated in GABA_B_R, it could come from the spontaneous closure of VFT, which is abolished by the competitive antagonist^33,59^. Alternatively, it could also originate from any other step following the closure of the VFT such as the CRD and 7TM dimer rearrangement^82^. As demonstrated in mGluR5, the constitutive activity could come from the 7TM domain that can reach an active state even if the VFTs remain in their inactive orientation^55^. GABA_B_R is an additional instance where a genetic mutation S695I in human GABA_B2_ 7TM could trigger strong constitutive activity, which was associated with epileptic encephalopathy^63^. As such, several specific structural determinants in the CaSR could be largely limit or even suppress its constitutive activity. This is in line with the identification of more than one hundred mutations in the ECD and 7TM domains of CaSR in patients with gain-of-function CaSR associated with ADH^39^. However, only a few of them, such as N802I in TM6 and A843E in TM7, have been investigated for their constitutive activity^83,84^.

In the present study, we examined the possible role of the subunit interface at the level of the VFT dimer, as it may regulate the basal activity of CaSR. We show that the inter-subunit disulfide bridges on the upper loops of LB1 is required to maintain a completely inactive state with low Ca^2+^ potency in the 2.2–2.6 mM range. This concentration range is optimal for the physiological function of the receptor in controlling calcemia. We uncovered that all natural mutations occurring at either C129 or C131 could trigger the constitutive activation of CaSR. Therefore, the integrity of two inter-subunit disulfide bridges is required to abolish constitutive activity of CaSR. This finding might provide an explanation for calcemia induced by mutations in either C129 or C131.

Constitutive internalization and constitutive activity are usually related, and could be blocked by reverse agonists. Previous studies demonstrated that CaSR undergoes constitutive and agonist induced internalization, both of which were G protein-independent and β-arrestin-dependent at least partially^85^. Interestingly, a negative allosteric modulator did not inhibit the constitutive internalization of CaSR, which suggests there is no link between basal activity and constitutive internalization of CaSR. However, it needs to be examined in more details in future studies.

Among the class C GPCRs examined in this study, only CaSR had no detectable constitutive activity, this observation may be attributed to the fact that CaSR has two inter-subunit bonds. These cysteine residues have been conserved from fish to mammals, illustrating their evolutionary importance and their ability to tightly control a very low basal activity. In contrast to what was observed for CaSR, the mGluRs and GPRC6A which only have one inter-subunit bridge, mutation of the unique inter-subunit disulfide bond in mGluR2 reduced the apparent agonist affinity and decreased the stability of the active state^4^. The distinct roles of inter-subunit disulfide bonds between mGluRs and CaSR may be due to the different sequence and conformation of the upper loop where the disulfide bridge is located, but also the preceding α-helix B which participates in the dimer interface between the two protomers. In mGluRs, this helix B adopts a different conformation between the inactive and active states. Interestingly, the loop immediately preceding it contains residues important for ligand recognition in the VFT binding pocket^86^. Agonist binding in the VFT can therefore trigger relaxation of the inter-subunit interface by acting on this helix B, which releases its constraint upon activation. Concerning the upper loop, its structure is often not solved in the class C GPCRs because of its flexibility^51,87–89^. However, in the few structures where this loop could be observed, its conformation in CaSR and mGluRs is different (Supplementary Fig. 10a–d). Another difference in the activation mechanism between CaSR and mGluRs, which could affect constitutive activity, is the large amplitude of VFT dimer reorientation observed for mGluRs between inactive and active states. In CaSR, the amplitude of VFT reorientation is not as large as in the mGluRs. Mutation of the inter-subunit disulfide bridge in mGluRs could limit this VFT reorientation, thus reducing constitutive activity as observed in our study for both mGluR2 and mGluR5. In brief, it is reasonable to presume that the inter-subunit disulfide bond in the upper loop may affect the receptor’s equilibrium between active and inactive state^1,2^, but additional experimental approaches and methods are required to fully investigate the precise mechanisms in the future.

Our findings with inter-subunit disulfide bridge mutants provide insights on the molecular mechanism of CaSR activation. Cryo-EM structures of CaSR confirmed that L-AAs binding stabilizes the closed state of VFT, an intermediate state which is more easily activated by Ca^2+^ but does not result in receptor activation^49^. We uncovered through mutagenesis that the basal activity induced by impairing the inter-subunit disulfide bond was most probably caused by the ambient L-AAs. And when the integrity of the inter-subunit covalent link was lost, L-AAs occupied only one VFT pocket were sufficient to drive the receptor activation. Removing the inter-subunit covalent link most likely decreases the activation energy barrier and enabling L-AAs binding to activate the receptor, as well as increasing the agonist potency of the receptor. This further validates the function of inter-subunit disulfide links in restricting the CaSR activation for better adaptation to complex environmental stimuli, and also explains why L-AAs have no agonist activity but behave as pure-PAMs^45^.

Our results on the LB2 interface also help to clarify the molecular mechanism of CaSR activation. This region with highly negatively charged residues is involved in the contact formation between LB2 domains upon the activation of class C GPCRs. Binding of cations, such as Gd^3+^, can neutralize the electrostatic repulsion in mGluRs and stabilize receptor active conformation^90^. The CaSR LB2 interface exhibits significantly higher electrostatic repulsion than mGluRs because it contains approximately twice as many negatively charged residues^45,58^. The electrostatic repulsion from this region was proposed to be able to allosterically control CaSR activation^58^. Our previous study demonstrated that mutations in this region did not alter the Ca^2+^ potency and excluded the possibility that this region has a functional binding site for Ca^2+^ ^45^. Here we show that neutralization of negatively charged residues in this region fails to change the Ca^2+^ potency, but significantly enhanced the agonist effect of PAMs on CaSR and the basal activity of the CSCS mutant. Therefore, we speculated that the inter-subunit covalent bridges has a stronger importance than the electrostatic repulsion caused by the LB2 interface to stabilize the inactive state. Disruption of the inter-subunit disulfide bridges could bring the two LB2 lobes into proximity to induce an activation signal. And this activation signal could be further facilitated by reducing the electrostatic repulsion at the LB2 interface. However, in presence of the inter-subunit disulfide bridges, only reducing the electrostatic repulsion in the receptor did not alter the calcium potency or basal activity. Overall, inter-subunit disulfide bridges are the main determinant limiting receptor activation by raising the energetic barrier to CaSR activation. Electrostatic repulsion at the LB2 interface is a secondary determinant that reinforces stabilization of the inactive state. The combination of these two determinants enables CaSR to accurately detect surrounding complex nutrient signals.

**Fig. 7:**
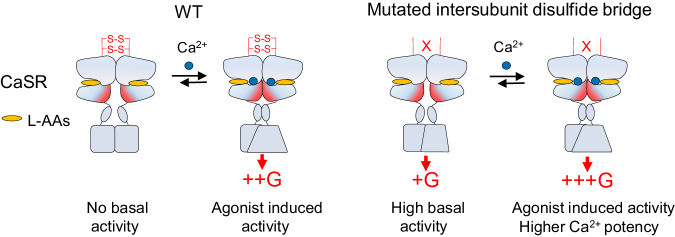
Model of CaSR activation. The cartoons highlight the role of inter-subunit disulfide bonds in negatively regulating CaSR activity and limiting its constitutive activity. Compared with WT, the inter-subunit disulfide bond mutants displays constitutive activity and higher potency to CaCl_2_.

Taken together, our study demonstrates that the specific structural framework of VFT provide molecular basis for the unique activation features of CaSR, permitting for fine tuning of the ligands sensitivity of the receptor. All results allowed us to propose a molecular lock model, which speculates that the inter-subunit disulfide bonds serve as molecular locks to restrict the receptor’s dynamics and stabilize it in inactive conformations (Fig. 7). Removal of the inter-subunit disulfide bonds enables L-AAs alone to activate CaSR and elevates the receptor’s agonist potency. Therefore, these findings highlight the importance of optimal constitutive activity of receptors and provide valuable insights for the design and development of targeted drugs.

## Methods

### Materials

Calcium chloride (Cat. 10043-52-4), magnesium chloride (Cat. 7786-30-3), GABA (Cat. A2129), L-glutamate (Cat. 56-86-0), L-alanine (Cat. 56-41-7), L-trytophan (Cat. 73-22-3), neomycin (Cat. 1405-10-3) and spermine (Cat. 71-44-3) were purchased from Sigma-Aldrich (St. Louis, MO, USA). NPS R-568 (Cat. 3815) and AC265347 (Cat. 6165) were purchased from Tocris Bioscience (Bristol, UK). NPS 2143 (Cat. ab145050) was obtained from Abcam (Cambridge, UK). Etelcalcetide (Cat. HY-P1955A) was from MedChemExpress (NJ, USA). Lipofectamine 2000 (Cat. 11668019) and Fluo4-AM (Cat. M14206) were supplied by Thermo Fisher Scientific (Waltham, MA, USA). SNAP-Surface 649 (Cat. S9159S) was from New England Biolabs (Ipswich, MA, USA). IP-One Gq kit (Cat. 62IPAPEB), BRET substrate coelenterazine h (Cat. S2001) and furimazine (Cat. N1120) were from Revvity (Codolet, France) and Promega (Madison, WI, USA), respectively.

### Plasmids and transfection

All the plasmids encoding the receptors CaSR, mGluR2, mGluR5, and GPRC6A have a SNAP-tag before the HA- or Flag-tag. The pRK5 plasmid encoding wild-type human CaSR, with Flag or HA and SNAP tags inserted after the signal peptide^45^. We have verified that the Flag and HA-tagged CaSR are similarly expressed on the cell surface (Supplementary Fig. 11a) and showed they have the same potency and E_max_ upon calcium stimulation (Supplementary Fig. 11b), under the same transfection conditions. The pRK5 plasmid encoding the rat mGluR2 or mGluR5, labeled with HA and SNAP inserted just after the signal peptide^73,82^. The pRK5 plasmid encoding rat GB1, tagged with HA inserted after the signal peptide, and the rat GB2, tagged with Flag inserted after the signal peptide were kindly provided by the Institut de Génomique Fonctionnelle (Montpellier, France)^63^. The pRK5 plasmid encoding wild-type human GPRC6A, with a Flag and SNAP tags inserted after the signal peptide was a gift from Revvity. For BRET saturation experiments, plasmids were generated by inserting the Rluc or YFP sequence at the C-terminus of ^Flag-SNAP^CaSR. The last 192 residues of CaSR C-terminus were replaced by the sequence coding C1KKXX (C1, the coiled-coil sequence of 47 residues at the C-terminus of GABA_B1_) or C2KKXX (C2, 49 residues of GABA_B2_ coiled-coil region, followed by the endoplasmic reticulum retention signal KKTN) to obtain the plasmids encoding ^HA-SNAP^CaSR_C1_ and ^Flag-SNAP^CaSR_C2_. The mutations for CaSR and mGluRs were generated by site-directed mutagenesis using the QuikChange mutagenesis protocol (Agilent Technologies). All constructs were verified by DNA sequencing.

All experiments were performed in HEK293 (ATCC, CRL-1573, lot: 3449904) cultured in DMEM media (Thermo Fisher Scientific) containing 10% fetal bovine serum (Thermo Fisher Scientific), 100 units/ml penicillin and 100 μg/ml streptomycin (Thermo Fisher Scientific) at 37 °C and 5% CO_2_. Cells were transiently transfected either by electroporation or using lipofectamine 2000 according to the manufacturer protocol (Invitrogen Life Technologies)^72^. In the electroporation transfection, ten million cells were transfected with a total of 4 μg plasmids. In the liposome transfection assay, two million cells were transfected with 1 μg of total amounts plasmid of interest. In order to couple mGluR2 and GABA_B_R to the phospholipase C pathway, the cells were co-transfected with the chimeric G protein Gqi_9_. For experiments involving mGluRs, the cells were also co-transfected with the glutamate transporter EAAC1. The ratio of amount plasmids for the constructs expressing the indicated receptor, Gqi_9_ and EAAC1 is 2:1:1.

### Enzyme-linked immunosorbent assay

Cell surface and total expression of the indicated constructs were quantified by an enzyme-linked immunosorbent assay (ELISA). Cell culture medium was removed from 96-well plates 24 h after transfection, then cells were washed and fixed with 4% paraformaldehyde (Sigma-Aldrich), then blocked with 10% FBS (for the total expression group, 0.1% Triton X-100 (Thermo Fisher Scientific) was required before this step to increase the permeability of the cell membrane). Flag-tagged constructs were detected with the monoclonal mouse anti-Flag antibody coupled with horseradish peroxidase (1:20,000; F1804, Sigma-Aldrich). HA-tagged constructs were detected with the rat anti-HA antibody coupled with horseradish peroxidase (1:20,000; 3F10, Roche, Indianapolis, USA). Bound antibodies were detected by chemoluminescence using a Super Signal substrate (Thermo Fisher Scientific) and a 2103 EnVision Multilabel Plate Reader (Perkin Elmer, Waltham, MA, USA).

### Intracellular calcium measurements

Intracellular Ca^2+^ release was measured in 96-well plates of HEK293 cells with a calcium-sensitive fluorescent dye (Fluo4-AM, Thermo Fisher Scientific)^45^. Briefly, 24 h after transfection, cells were washed and pre-incubated with 1 μM Fluo-4 AM in Flex buffer (containing 130 mM NaCl, 5.1 mM KCl, 0.42 mM KH_2_PO_4_, 0.32 mM Na_2_HPO_4_, 5.27 mM glucose, 20 mM HEPES, 3.3 mM Na_2_CO_3_, 0.1% BSA, 2.5 mM probenecid, pH 7.4) at 37 °C for 1 h. Then, cells were washed once with Flex buffer and 50 μl of this buffer was added into the wells. And 50 μl of the indicated compounds at 2-fold final concentrations were injected at 20 s during 60 s recording. Fluorescence signals (excitation 485 nm, emission 525 nm) were measured by using the fluorescence microplate reader Flexstation 3 (Molecular Devices, Sunnyvale, CA, USA). The Ca^2+^ response is given as the agonist-stimulated fluorescence increase.

### Inositol phosphate (IP_1_) measurements

IP_1_ accumulation was determined in 96-well plates using the IP-One HTRF kit (62IPAPEJ, Revvity) according to the manufacturer’s instructions. Briefly, 24 h after transfection, cells were washed and incubated with indicated compounds in the Ca^2+^-free stimulation buffer (containing 10 mM HEPES, 146 mM NaCl, 4.2 mM KCl, 1 g/L glucose, 50 mM LiCl, pH 7.4) at 37 °C for 30 min. The d2-labeled IP_1_ (IP_1_-d2) and terbium cryptate-labeled anti-IP_1_ antibody (Anti-IP_1_-K) were diluted in lysis buffer (provided by the IP-One HTRF kit) and added in each well, then the plate was incubated in the dark at room temperature for 1 h before detected by the Multi-mode plate reader (PHERAstar FSX, BMG LABTECH). Results were calculated from the standard curve in the kit with the fluorescence ratio of IP_1_-d2 emission at 665 nm over the Anti-IP_1_-K emission at 620 nm. For the experimental group of L-AAs elution and rebinding treatments, the Mg^2+^ and Ca^2+^ free HBS starvation buffer (containing 10 mM HEPES, 140 mM NaCl, 4 mM KCl, 1 mM KH_2_PO_4_, pH 7.4) was used to wash cells for three times during 3 h, then the cells were incubated with the indicated compounds in the stimulation buffer.

### Bioluminescence resonance energy transfer (BRET)

To measure the dimerization of CaSR WT and the indicated mutants, two million cells were co-transfected with a constant amount of Rluc-tagged receptors (0.2 μg) and an increasing amount of YFP-tagged receptors (0, 0.05, 0.1, 0.2, 0.4, 0.6, 0.8, and 1 μg) and completed to a total amount of 1.2 μg with the plasmid encoding the pRK5 empty vector. Cell culture medium was removed from 96-well plates 24 h after transfection, then cells were washed and incubated in HBS buffer (containing 10 mM HEPES, 140 mM NaCl, 4 mM KCl, 1 mM KH_2_PO_4_, pH 7.4) at 37 °C. YFP fluorescence was measured before the addition of Rluc substrate coelenterazine H. Then each well was loaded with 40 μL HBS with coelenterazine H at a final concentration of 5 μM, and the signal was read by a Mithras LB 940 (Berthold Biotechnologies, Bad Wildbad, Germany) at 37 °C for six cycles. The net BRET was defined as the experimental BRET signal values with the baseline subtracted.

For G protein activation experiments, two million cells were transfected with 0.5 μg receptor plasmid, 0.02 μg the indicated Gα fused to Nluc, 0.2 μg Gβ_1_ and 0.2 μg Venus-tagged Gγ_9_ by Lipofectamine 2000. Cell culture medium was removed from 96-well plates 24 h after transfection, then cells were washed and each well was loaded with 40 μL of the Nluc substrate furimazine at a final concentration of 10 μM. After measuring the baseline BRET signal, cells were treated with 10 μL of drugs (prepared in HBS at 5-fold final concentration) for an additional 6 cycles. Results were calculated as the ratio of the Venus emission at 485 nm over the Nluc emission at 530 nm.

### Western blot

Twenty-four hours after transfection, cells were washed and incubated with or without the indicated treatments in HBS buffer at 37 °C for 2 h. Then the cells were lysed with ice-cold lysis buffer (containing 50 mM Tris-HCl pH 7.4, 150 mM NaCl, 1% Triton X-100, 1% sodium deoxycholate, 1% SDS), supplemented with complete phosphatase inhibitor cocktail (Roche) and incubated on ice for 30 min. Samples were denatured with loading buffer (containing 250 mM Tris-HCl pH 6.8, 50% (v/v) glycerol, 10% (w/v) SDS, 0.5% (w/v) bromophenol blue) and 10 mM DTT for 10 min at 95 °C, separated by SDS-PAGE (10% w/v), and then transferred to nitrocellulose membranes (Millipore, Billerica, MA, USA) and washed with blocking buffer (5% nonfat dry milk in Tris-buffered saline and 0.1% Tween 20) for 2 h at 25 °C. The following primary antibodies were used for western blot analysis: polyclonal anti-p44/42 ERK and anti-phospho-p44/42 ERK antibodies (1:3000; 9101 and 9102, Cell Signaling Technology, Shanghai, China), monoclonal Flag antibody (1:1000; KM8002, Sungene Biotech, Tianjin Province, China) and polyclonal anti-α-tubulin antibody (1:3000; KM9007, Sungene Biotech, Tianjin Province, China). The primary antibodies were incubated overnight at 4 °C, followed by incubation with the DyLight 800 4 X PEG-conjugated secondary antibody (1:20,000; 5151 and 5257, Cell Signaling Technology, Shanghai, China) for 2 h at 25 °C. The specific protein bands were visualized by Odyssey CLx imager (LI-COR Bioscience, Lincoln, NE, USA). The density of bands was measured by ImageJ software (Bethesda, MD, USA).

### Fluorescent-labeled blot experiments

The dimerization pattern of CaSR mutants were measured by fluorescent-labeled blot^57,63^. Briefly, 24 h after transfection, adherent HEK293 cells in 12-well plates were incubated with 100 nM SNAP-Surface 649 in culture medium in the dark at 37  °C for 1 h, and then lysed with lysis buffer (containing 50 mM Tris-HCl pH 7.4, 150 mM NaCl, 1% NP-40, 0.5% sodium deoxycholate, 0.1% SDS) for 1 h at 4 °C. After centrifugation at 12,000 × *g* for 30 min at 4 °C, the supernatants were mixed with loading buffer (containing 250 mM Tris-HCl pH 6.8, 50% (v/v) glycerol, 10% (w/v) SDS, 0.5% (w/v) bromophenol blue) at 37 °C for 10 min and separated by SDS-PAGE (8% w/v). In reducing conditions, samples were treated with 100 mM DTT in loading buffer for 10 min before loading the samples. Proteins were transferred to nitrocellulose membranes (Millipore). The specific protein bands were visualized by Odyssey CLx imager (LI-COR Bioscience).

### Reverse transcription PCR and real-time PCR

Total RNA of mock and CaSR-overexpressed cells were extracted using Trizol reagent (Invitrogen, Carlsbad, USA) and reverse transcribed into cDNA using the Superscript first-strand synthesis system (Invitrogen). *GAPDH* and *CASR* mRNA expressions were examined. The primers used were as follows: *GAPDH* (NM_001256799), 5′-tcaccagggctgcttttaacc-3′ and 5′-gacaagcttcccgttctcag-3′; *CASR* (NM_000388), 5′-ccctctacgattgctgtggt-3′ and 5′-agtctgctggaggaggcata-3′.

Real-time PCR was conducted using the SYBR Green (Vazyme Biotechnology, Nanjing, China) and the StepOne plus Real-time PCR System (Thermo Fisher Scientific). The threshold was set according to the exponential phase of products, and the cycle threshold (CT) value for samples was determined. The resulting data were analyzed with the comparative CT method for relative gene-expression quantification against *GAPDH*.

### Molecular modeling

The structures of the receptors involved were analyzed and displayed with PyMol software (Palo Alto, CA, USA). The molecular models were generated with PyMol software based on the structures from the PDB (7DTV, 7DTW, 5FBK, 7M3E, 7M3G, 7M3J and 7M3F for CaSR, 7EPA and 7EPB for mGluR2, 7FD8 for mGluR5). The sequence of CaSR between different species or the human CaSR and different rat mGluR subtypes were aligned with Clustal Omega and displayed by ESPript 3^91^.

### Statistics and reproducibility

Data were analyzed with Prism 7 software (GraphPad Software, San Diego, CA, USA). Concentration-responses curve parameters were derived using a four parameters non-linear regression equation. Unless stated otherwise, data shown in the figures represent the mean ± S.E.M. of at least three independent experiments performed in triplicates. Statistical differences were determined by GraphPad Prism using one-way ANOVA with a Dunnett’s multiple comparison test, two-way ANOVA with Sidak’s multiple comparisons test or t test. *P* < 0.05 was considered to be statistically significant.

### Supplementary information

Supplementary Information

Description of Additional Supplementary Files

Supplementary Data

reporting-summary

## Data Availability

Data supporting the findings of this manuscript are available from the corresponding authors upon reasonable request. The source data underlying the graphs in the manuscript are shown in Supplementary Data. The uncropped unedited blot/gel images were shown in Supplementary Figs. 12 and 13.
