# Supplementary material for: Absence of calcium-sensing receptor basal activity due to inter-subunit disulfide bridges
Source: Commun Biol. 2024 Apr 25;7:501. doi: 10.1038/s42003-024-06189-3 (PMC11045811; doi:10.1038/s42003-024-06189-3)
Supplement: Supplementary file 1 — Supplementary Information [file 42003_2024_6189_MOESM1_ESM.pdf]

# SUPPLEMENTARY INFORMATION

## Absence of calcium-sensing receptor basal activity due to inter-subunit disulfide bridges

Shumin Ma[1], Xueliang Yin[1], Jean-Philippe Pin[2], Philippe Rondard[2]*, Ping Yi[1]*, Jianfeng Liu[1]*

[1] Cellular Signaling Laboratory, International Research Center for Sensory Biology and Technology of MOST, Key Laboratory of Molecular Biophysics of MOE, and College of Life Science and Technology, Huazhong University of Science and Technology, 430074 Wuhan, Hubei, China.

[2] Institut de Génomique Fonctionnelle (IGF), Université de Montpellier, CNRS, INSERM, 34094 Montpellier Cedex 5, France.

* Philippe Rondard, Ping Yi, Jianfeng Liu

**Email:** jfliu@mail.hust.edu.cn; pingy@hust.edu.cn; philippe.rondard@igf.cnrs.fr

**This PDF file includes:**
Supplementary Figure 1-13.

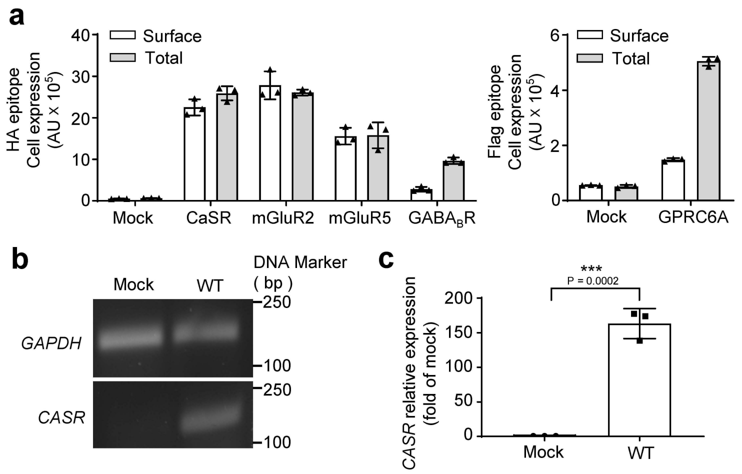


**Supplementary Figure 1. Expression analysis of CaSR and the different class C GPCRs. (a)** Surface and total cell expression levels of the indicated receptors. Amounts of HA-tagged or Flag-tagged receptors at the cell surface and in the permeabilized cells were quantified by ELISA. Data are mean ± SD from a typical experiment performed in triplicates (n = 3). **(b)** Detection by electrophoresis of *CASR* and *GAPDH* (positive control) cDNAs from the total mRNA expression in HEK293 cells transfected with human CaSR or an empty plasmid (Mock) after RT-PCR. Results are representative of a typical experiment (n = 3). Uncropped blots are presented in the Supplementary Figure 12. **(c)** *CASR* expression in HEK293 cells transfected with CaSR or empty plasmid (Mock) was quantified by real-time qPCR. Quantitative data are mean ± SEM of three independent experiments performed in triplicates. Significance was analyzed using t test with ***P ⩽ 0.001 versus the mock.

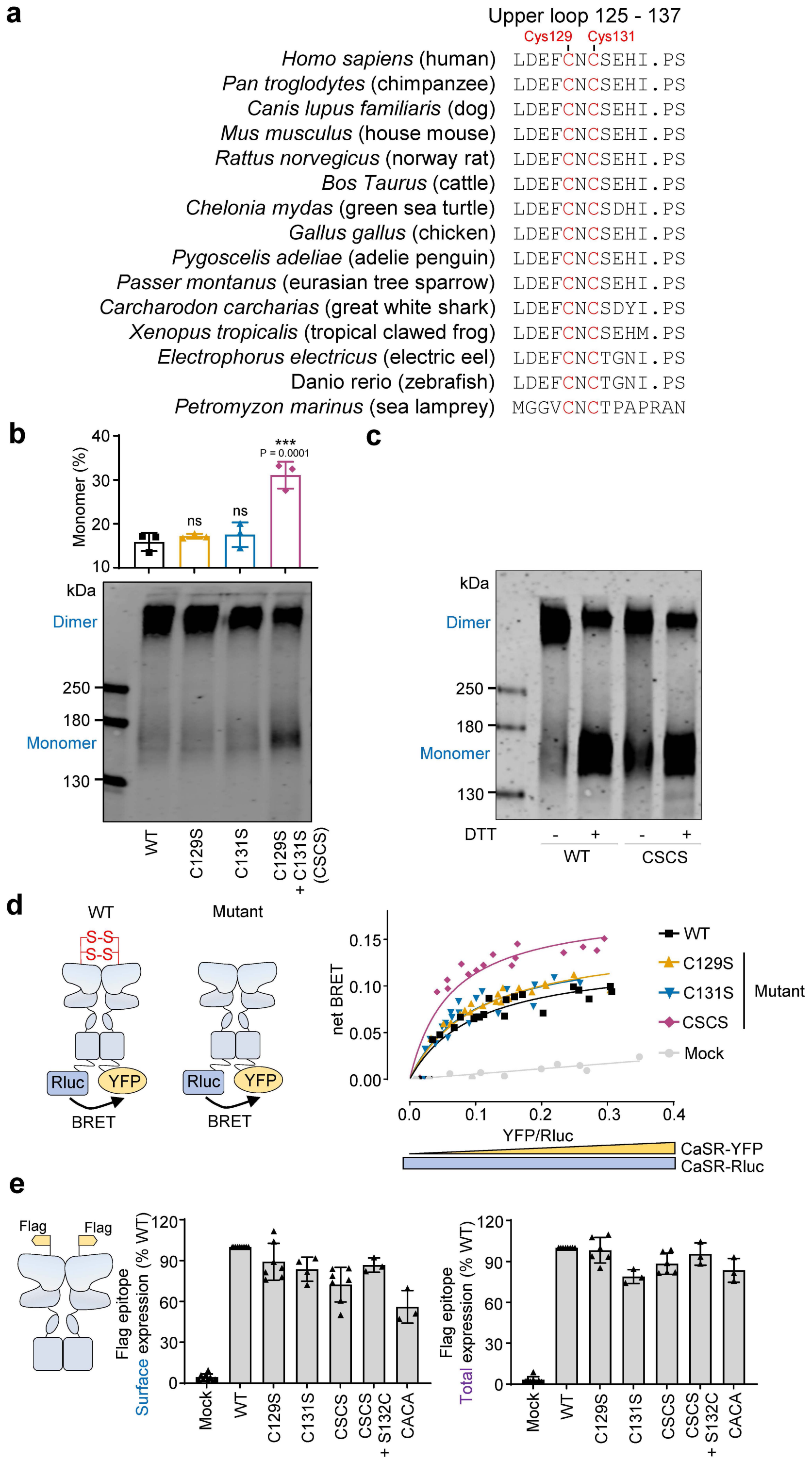


**Supplementary Figure 2. Deletion of the inter-subunit disulfide bonds leads to basal activation of CaSR. (a)** Sequence alignment of the upper loop in different species of CaSR by Clustal Omega and ESPript 3. Human CaSR was used reference for residue numbering.

**(b)** SDS-PAGE analysis of cell surface SNAP-tagged CaSR subunits of the WT and indicated mutants under non-reducing conditions after labelling of the cell surface receptors with a non-permeant fluorescent SNAP substrate. Changes of monomer ratio are quantified (n = 3). Uncropped blots are presented in the Supplementary Figure 12. **(c)** SDS-PAGE analysis of cell surface SNAP-tagged CaSR subunits of the WT and CSCS under non-reducing conditions or reducing conditions (10 mM DTT) after labelling of the cell surface receptors with a non-permeant fluorescent SNAP substrate (n = 3). Uncropped blots are presented in the Supplementary Figure 12. **(d)** Cartoon illustrating the BRET sensor used to detect CaSR dimer formation. BRET signal was measured in HEK293 cells co-transfected with a fixed amount of CaSR-Rluc and increasing amounts of CaSR-YFP, and plotted against the ratio of YFP emission (before substrate addition) to Rluc luminescence. Net BRET was calculated by the BRET ratio corrected by the baseline BRET signal obtained in cells transfected only with CaSR-Rluc. The two constructs were transfected as shown in the schemes, either with wild-type (WT) YFP and Rluc-tagged constructs, or with both mutated constructs (n = 3). **(e)** Surface and total cell levels of the CaSR mutants. Amounts of Flag-tagged CaSR mutants at the cell surface and in the permeabilized cells were quantified by ELISA (n = 3-7). Data above are mean ± SEM of at least three independent experiments performed in triplicates. Blots is representative of a typical experiment. Significance was analyzed using one-way ANOVA with Dunnett's multiple comparisons with *** P $\leqslant$ 0.001 and ns for P > 0.05 versus the WT.

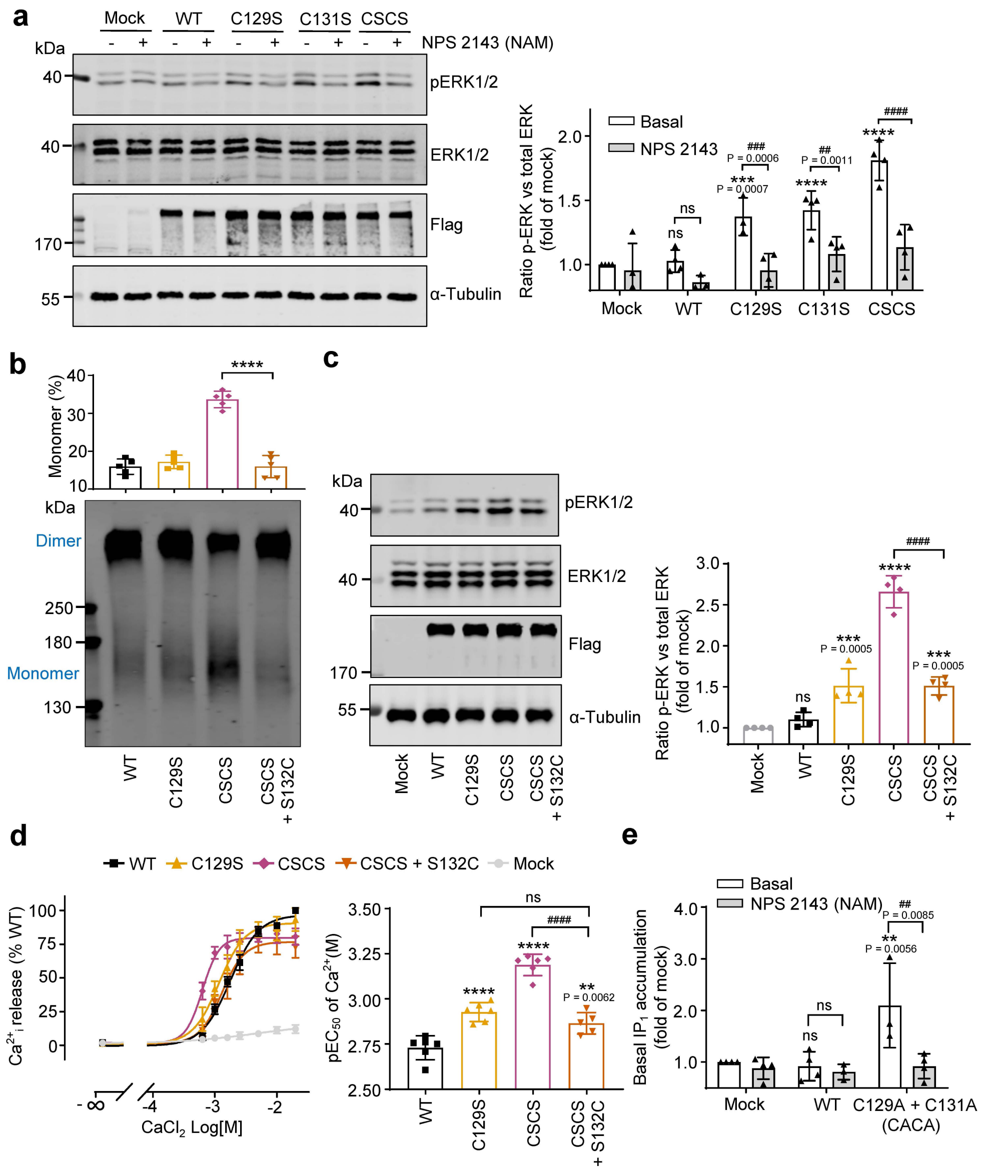


**Supplementary Figure 3. Reintroducing an inter-subunit disulfide bridge reduce the basal activity of CSCS. (a)** ERK1/2 activation measurement for the WT and indicated mutants treated with or without NAM NPS-2143 (10 μM for 2 hours). Changes of ERK1/2 phosphorylation level were quantified (n = 4). Uncropped blots are presented in the Supplementary Figure 13. **(b)** SDS-PAGE analysis of cell surface SNAP-tagged CaSR subunits of the WT and indicated mutants under nonreducing conditions after labelling of the cell surface receptors with a non-permeant fluorescent SNAP substrate. Changes of monomer ratio are quantified (n = 5). Uncropped blots are presented in the Supplementary Figure 13. **(c)** Basal ERK1/2 activation for the WT and indicated mutants. Changes of ERK1/2 phosphorylation level were quantified (n = 5). Uncropped blots are presented in the Supplementary Figure 13. **(d)** Intracellular calcium release in the WT and indicated mutants treated with $CaCl_2$ and the corresponding $pEC_{50}$ (n = 6). **(e)** Basal $IP_1$ accumulation

measured for the WT and indicated mutants treated with or without NAM NPS-2143 (10 μM for 2 hours) (n = 4). Data above are mean ± SEM of at least four independent experiments performed in triplicates. Blots are representative of a typical experiment. Significance was analyzed using two-way ANOVA with Sidak's multiple comparisons (*a, e*) and one-way ANOVA with Dunnett's multiple comparisons (*b-d*) with **** P ⩽ 0.0001, *** P ⩽ 0.001, ** P ⩽ 0.01 and ns P > 0.05 versus the mock (*a, c*) or WT (*d*) and #### P ⩽ 0.0001, ## P ⩽ 0.01, # P ⩽ 0.005, and ns for P > 0.05 compared with indicated groups.

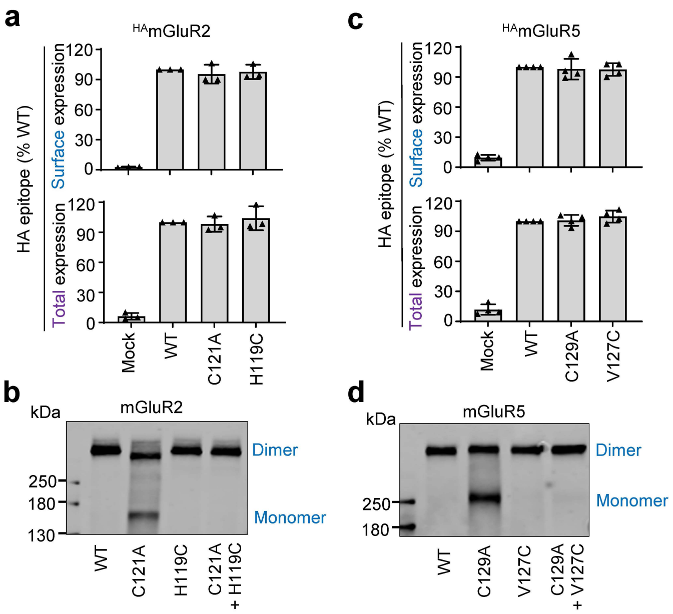


**Supplementary Figure 4. Expression analysis of mGluRs WT and mutants. (a, c)** Surface and total cell levels of the mGluR mutants. Amounts of HA-tagged mGluR2 (*a*, n = 3) and mGluR5 (*c*, n = 4) mutants at the cell surface and in the permeabilized cells were quantified by ELISA. **(b, d)** SDS-PAGE analysis of cell surface SNAP-tagged mGluR subunits for the WT and the indicated mutants under non-reducing conditions after labelling of the cell surface receptors with a non-permeant fluorescent SNAP substrate (n = 3). Data above are mean ± SEM of at least three independent experiments performed in triplicates and normalized to WT. Blots are representative of a typical experiment.

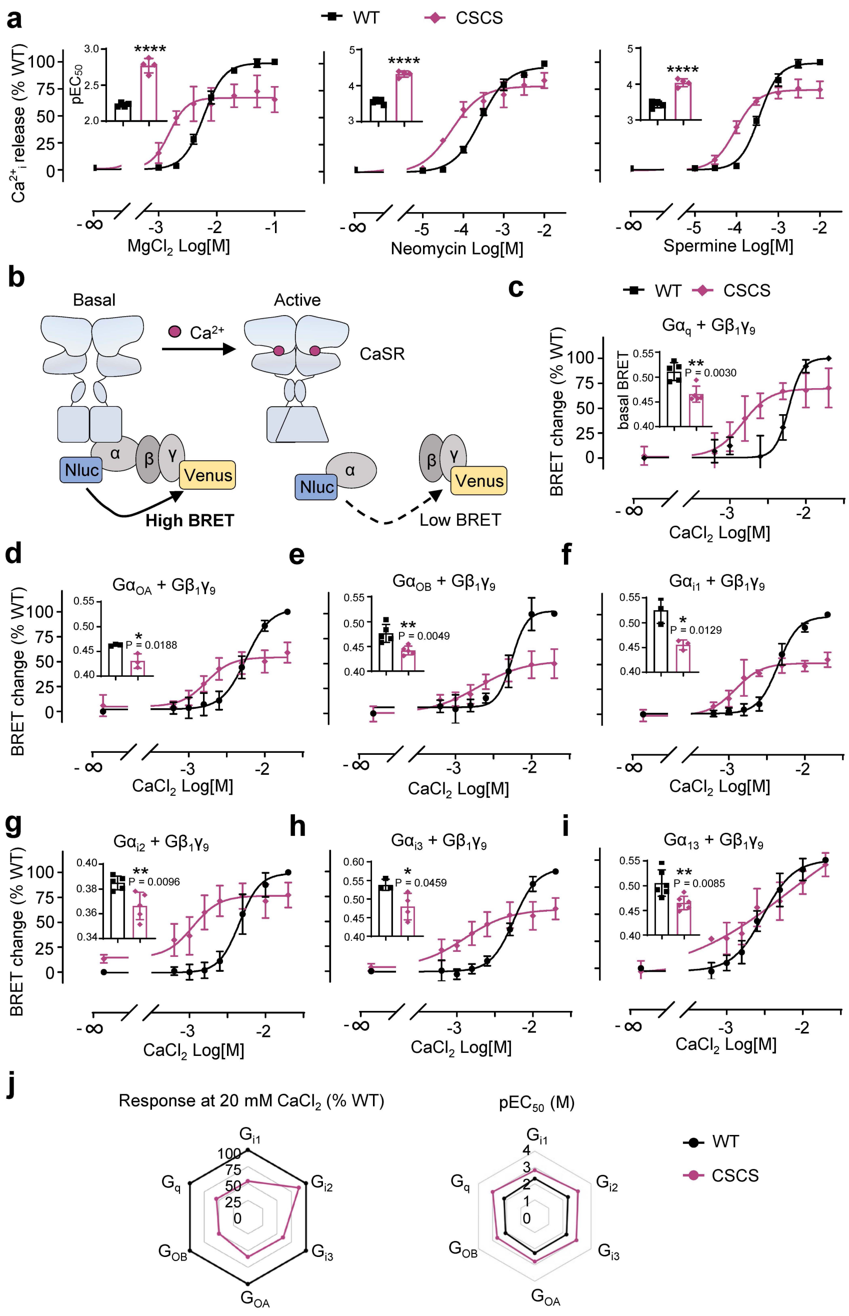


**Supplementary Figure 5. Pharmacology and G protein dissociation of CaSR WT and CSCS. (a)** Intracellular calcium release induced by the indicated agonists in WT and CSCS with the corresponding $pEC_{50}$ (inset) ($MgCl_2$, n = 4; neomycin, n = 5; spermine, n = 4). **(b)** Cartoon illustrating the BRET-based G protein sensor. **(c - i)** BRET ratio changes induced by $CaCl_2$ in the WT and CSCS, both co-expressed with $G\beta_1$, $G\gamma_9$-Venus and the indicated $G\alpha$-Nluc subunits (*c*, n = 7; *d*, n = 4; *e*, n = 8; *f*, n = 4; *g-i*, n = 6). The basal BRET ratio are shown in the inserted graphs. **(j)** Plots summarizing the activation of the indicated G proteins, in response to 20 mM $CaCl_2$ and $pEC_{50}$, by the wild-type CaSR receptor and the

CSCS mutant. Data above are mean ± SEM of at least four independent experiments performed in triplicates and normalized to the maximal response of WT. Significance was analyzed using t test with **** $P \leqslant 0.0001$, ** $P \leqslant 0.01$ and * $P \leqslant 0.05$ versus WT.

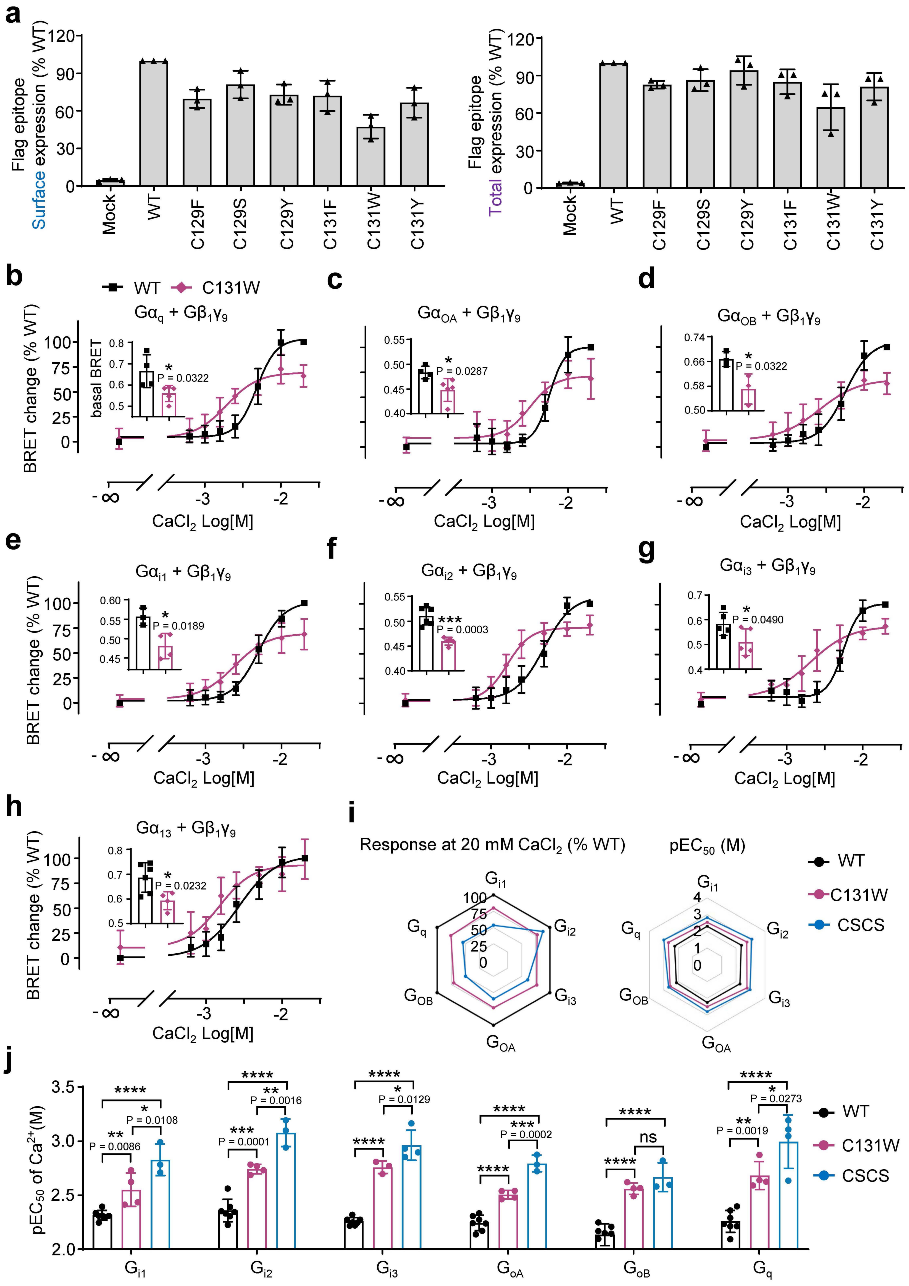

**Supplementary Figure 6. Characteristics of G protein dissociation of CaSR WT and C131W. (a)** Surface and total cell expression levels of the CaSR mutants. Amounts of Flag-tagged CaSR mutants at the cell surface and in the permeabilized cells were quantified by ELISA (n = 3). **(b - h)** BRET ratio changes induced by $CaCl_2$ in WT and C131W, both co-expressed with $G\beta_1$, $G\gamma_9$-Venus and the indicated Gα-Nluc subunits (*b*, n = 8; *c*, n = 7; *d*, n = 6; *e*, n = 5; *f*, n = 7; *g*, n = 4; *h*, n = 7). The basal BRET ratio are shown in the inserted graphs. **(i)** Plots summarizing the activation of the indicated G proteins, in response to 20 mM $CaCl_2$ and $pEC_{50}$, by the wild-type (WT) CaSR receptor and the C131W and CSCS mutants. The CSCS data are taken from the Supplementary Figure 5j. **(j)** Statistical analysis

of $pEC_{50}$ values for calcium stimulation measured in *(i)*. Data above are mean ± SEM of at least four independent experiments performed in triplicates and normalized to the maximum response of WT. Significance was analyzed using t test (*b-h*) or one-way ANOVA with Dunnett's multiple comparisons (*j*) with **** $P \leqslant 0.0001$, *** $P \leqslant 0.001$, ** $P \leqslant 0.01$ and * $P \leqslant 0.05$ versus the WT (*b-h*) or compared with indicated group (*j*).

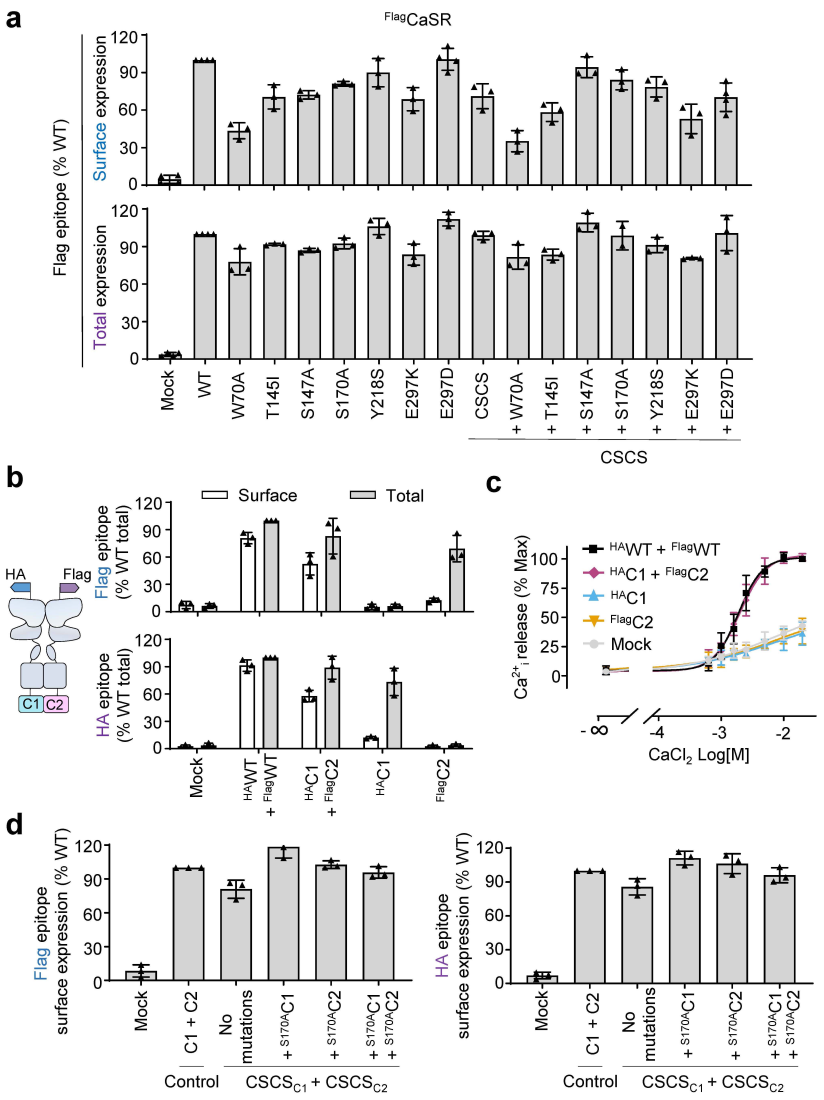


**Supplementary Figure 7. Expression and functional characterization of the controlled homodimer CaSR$_{C1C2}$.** **(a)** Surface and total cell expression levels of the CaSR mutants. Amounts of Flag-tagged CaSR mutants at the cell surface and in the permeabilized cells were quantified by ELISA (n = 4). **(b)** Cartoon illustrating the controlled homodimer formed by the constructs HA-tagged CaSR$_{C1}$ and Flag-tagged CaSR$_{C2}$. Quantification of cell surface and total expression of the indicated construct combinations by ELISA (n = 3) **(c)** Intracellular calcium release in the indicated subunit combinations stimulated with $CaCl_2$ (n = 4). **(d)** ELISA assay for cell surface and total expression of the indicated subunit combinations. (n = 4). Data above are mean ± SEM of at least three independent experiments performed in triplicates and normalized to WT (*a,b*) or the maximum response (*c*) or the control group (*d*).

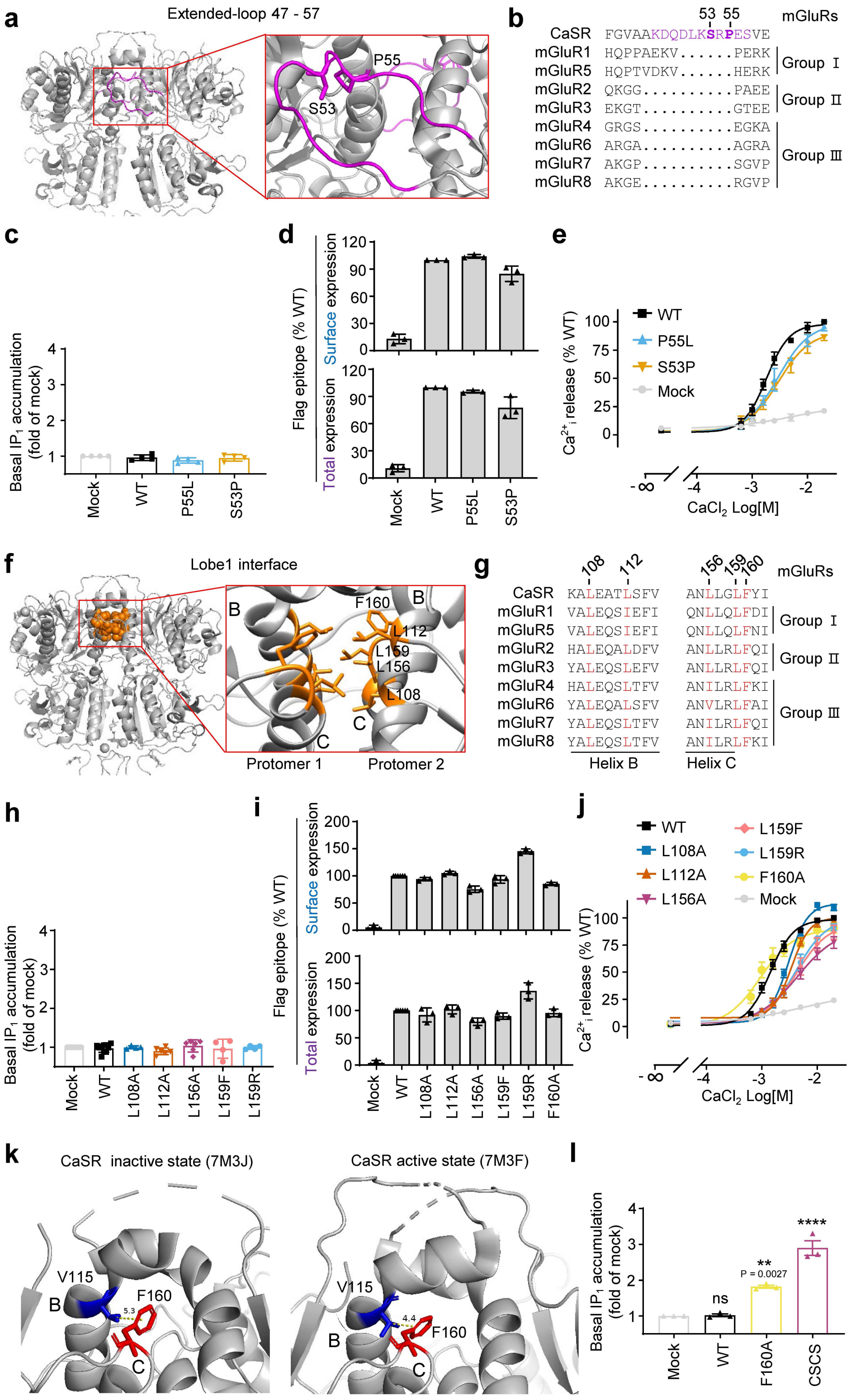


**Supplementary Figure 8. Upper interface of the VFT is not involved in CaSR basal activity. (a)** The extended loop (in purple) in the human CaSR structure (PDB: 7M3E).

Close-up view of the extended loop in one protomer. **(b)** Sequence alignment of the extended loop in human CaSR and rat mGluRs using Clustal Omega and ESPript 3. **(c)** Basal $IP_1$ accumulation in the WT and indicated mutants (n = 4). **(d)** Surface and total cell expression levels of the CaSR mutants. Amounts of Flag-tagged CaSR mutants at the cell surface and in the permeabilized cells were quantified by ELISA (n = 3). **(e)** Intracellular calcium release mediated by the indicated mutants upon stimulation with $CaCl_2$ (n = 3). **(f)** Interface between the two LB1 lobes in the human CaSR structure (PDB: 7M3E). Close-up view showing hydrophobic amino acid residues in the interface between the two LB1 lobes. **(g)** Sequence alignment of the LB1 interface in human CaSR and rat mGluRs by Clustal Omega and ESPript 3. **(h)** Basal $IP_1$ accumulation in the WT and indicated mutants. (n = 4 to 6). **(i)** Surface and total cell expression levels of the CaSR mutants. Amounts of Flag-tagged CaSR mutants at the cell surface and in the permeabilized cells were quantified by ELISA (n = 3). **(j)** Intracellular calcium release mediated by the indicated mutants upon stimulation with $CaCl_2$ (n = 3 to 4). **(k)** Residue Phe160 (F160) in the inactive and active state of the indicated human CaSR structures. **(l)** Basal $IP_1$ accumulation of the CaSR WT and the F160A and CSCS mutants (n = 3). Data above are mean ± SEM of at least three independent experiments performed in triplicates and normalized to the mock (*c, h, l*) or the WT (*d, e, i, j*). Significance was analyzed using one-way ANOVA with Dunnett's multiple comparisons with **** $P \leqslant 0.0001$, ** $P \leqslant 0.01$ and ns for $P > 0.05$ versus the mock.

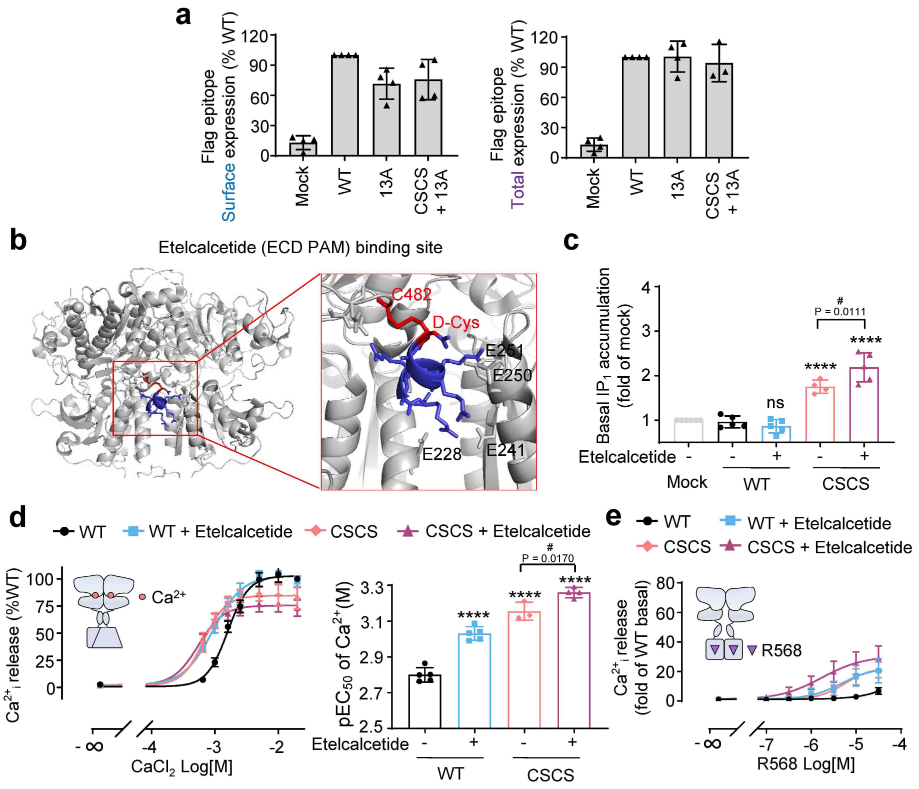

**Supplementary Figure 9. The expression of 13A mutant and the effect of ECD PAM etelcalcetide on CaSR activity. (a)** Surface and total cell levels of the CaSR mutants. Amounts of Flag-tagged CaSR mutants at the cell surface and in the permeabilized cells were quantified by ELISA (n = 4). **(b)** Etelcalcetide binding site shown in the CaSR structure (PDB: 7M3G). Etelcalcetide is shown in blue and forms a disulfide bond with its D-Cys (red) to the free CaSR Cys residue C482. The side chains of some CaSR glutamate residues interacting with etelcalcetide are highlighted. **(c)** Basal $IP_1$ accumulation in the WT and CSCS stimulated with or without 5 μM etelcalcetide (n = 5). **(d)** Intracellular calcium release mediated by the WT and CSCS mutant with or without etelcalcetide (5 μM) treatment upon stimulation with $CaCl_2$ and the corresponding $pEC_{50}$ (n = 5). **(e)** Intracellular calcium release for the WT and CSCS mutant stimulated by PAM R568 with or without etelcalcetide (5 μM) treatment (n = 4 to 5). Data above are mean ± SEM of at least four independent experiments performed in triplicates and normalized to the WT or the mock. Significance was analyzed using one-way ANOVA with Dunnett's multiple comparisons with **** P ⩽ 0.0001 versus WT, and # P ⩽ 0.05 versus CSCS.

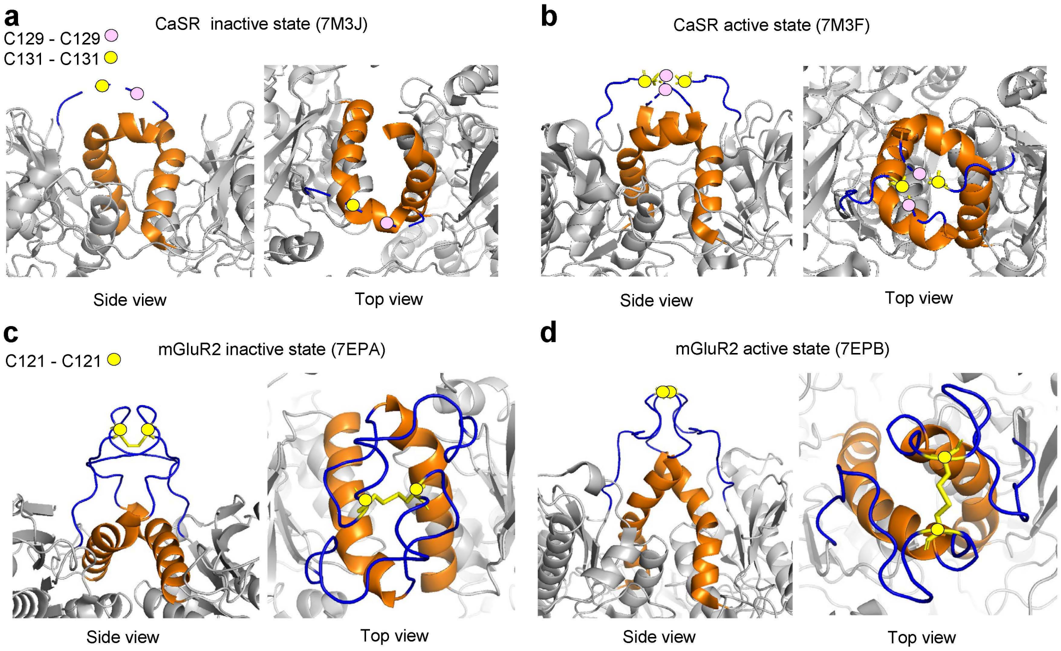


**Supplementary Figure 10. Structures of the upper loop and helix B of CaSR and mGluR2 in the inactive and active state.** Upper loop (blue) and helix B (orange) structures of CaSR and mGluR2 in the inactive and active states. Inter-subunit disulfide bonds, involving C129 and C131 for CaSR and C121 for mGluR2, are highlighted by colored circles.

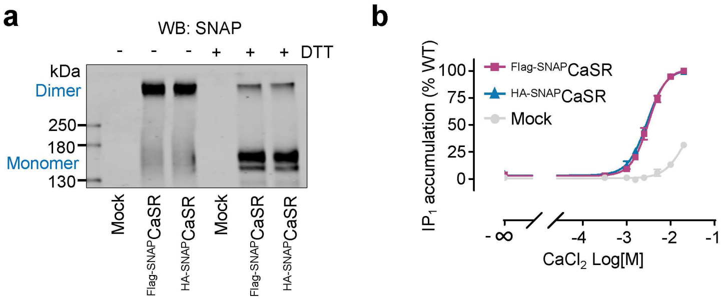


**Supplementary Figure 11. Expression and function analysis of Flag- or HA-tagged $^{SNAP}$CaSR-WT. (a)** Amount of cell surface Flag- or HA-tagged $^{SNAP}$CaSR-WT analyzed by blotting, under non-reducing or reducing conditions (10 mM DTT) after labelling of the cell surface receptors with a non-permeant fluorescent SNAP substrate. Blots are representative of a typical experiment (n = 3). **(b)** $IP_1$ accumulation induced by $CaCl_2$ in HEK-293 cells transfected with Flag- or HA-tagged $^{SNAP}$CaSR-WT. Data are mean ± SEM of three independent experiments performed in triplicates and normalized to the maximum response of Flag-tagged $^{SNAP}$CaSR-WT.

## Supplementary Figure 1b

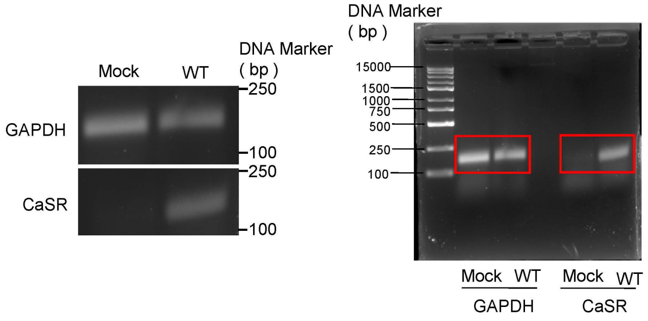


## Supplementary Figure 2b

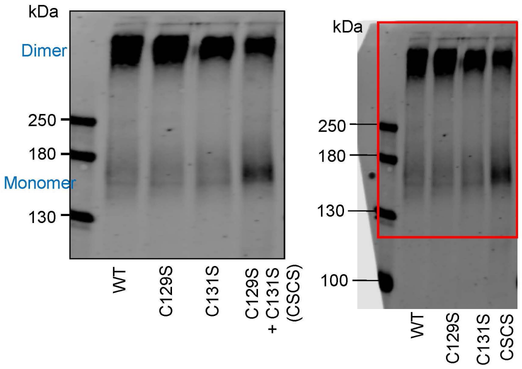


## Supplementary Figure 2c

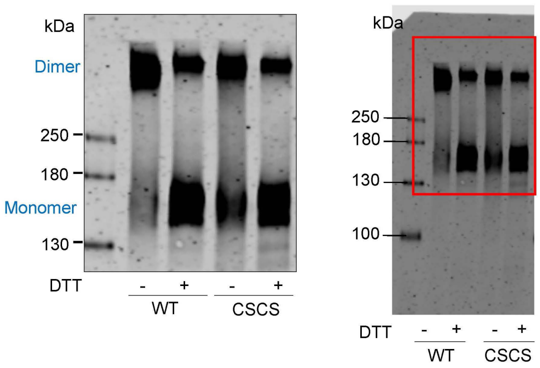


**Supplementary Figure 12.** Uncropped and unedited blot/gel images from supplementary Figure 1 and 2.

## Supplementary Figure 3a

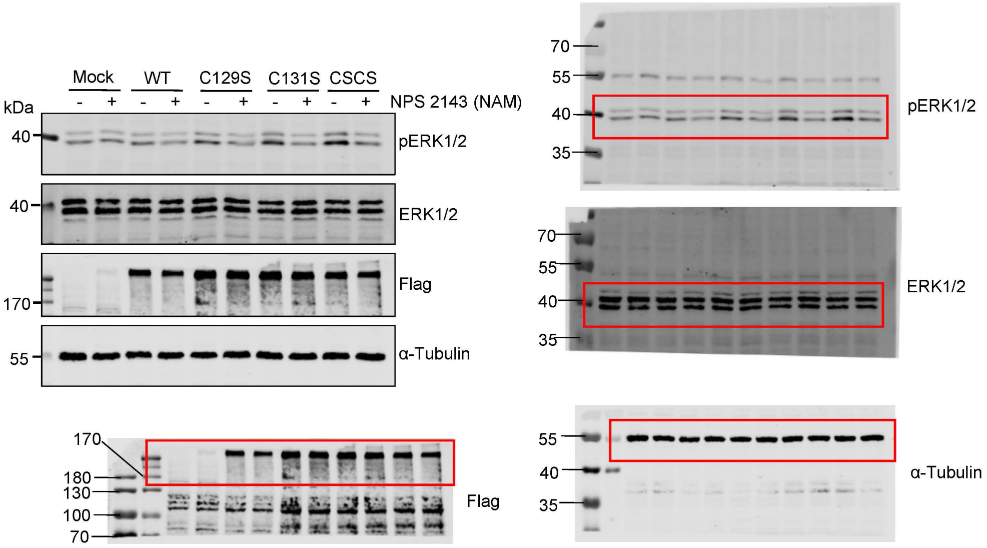


## Supplementary Figure 3b

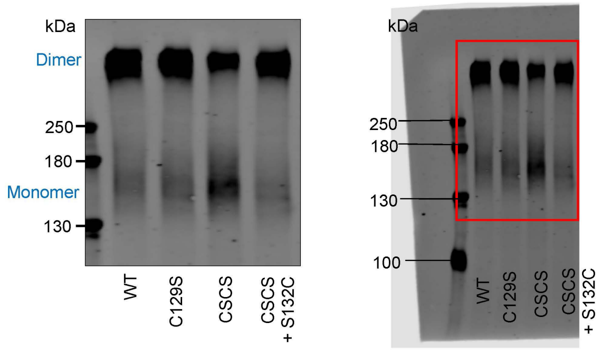


## Supplementary Figure 3c

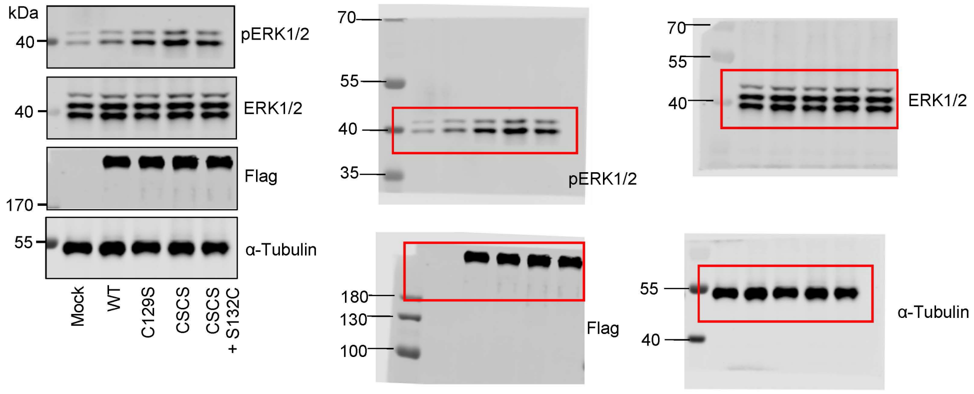


**Supplementary Figure 13.** uncropped unedited blot/gel images from supplementary Figure 3.
