# Supplementary material for: Absence of calcium-sensing receptor basal activity due to inter-subunit disulfide bridges
Source: Commun Biol. 2024 Apr 25;7:501. doi: 10.1038/s42003-024-06189-3 (PMC11045811; doi:10.1038/s42003-024-06189-3)
Supplement: Supplementary file 2 — Description of Additional Supplementary Files [file 42003_2024_6189_MOESM2_ESM.pdf]

Description of Additional Supplementary Files

File name: Supplementary Data

Description: The source data underlying the graphs in the manuscript.
