# Supplementary material for: Absence of calcium-sensing receptor basal activity due to inter-subunit disulfide bridges
Source: Commun Biol. 2024 Apr 25;7:501. doi: 10.1038/s42003-024-06189-3 (PMC11045811; doi:10.1038/s42003-024-06189-3)
Supplement: Supplementary file 4 — reporting-summary [file 42003_2024_6189_MOESM4_ESM.pdf]

nature portfolio

Corresponding author(s): Philippe Rondard, Ping Yi, Jianfeng Liu

Last updated by author(s): Mar 15, 2024

Reporting Summary

Nature Portfolio wishes to improve the reproducibility of the work that we publish. This form provides structure for consistency and transparency in reporting. For further information on Nature Portfolio policies, see our Editorial Policies and the Editorial Policy Checklist.

Statistics

For all statistical analyses, confirm that the following items are present in the figure legend, table legend, main text, or Methods section.

n/a	Confirmed	
☐	☒	The exact sample size (*n*) for each experimental group/condition, given as a discrete number and unit of measurement
☐	☒	A statement on whether measurements were taken from distinct samples or whether the same sample was measured repeatedly
☐	☒	The statistical test(s) used AND whether they are one- or two-sided<br>*Only common tests should be described solely by name; describe more complex techniques in the Methods section.*
☒	☐	A description of all covariates tested
☐	☒	A description of any assumptions or corrections, such as tests of normality and adjustment for multiple comparisons
☐	☒	A full description of the statistical parameters including central tendency (e.g. means) or other basic estimates (e.g. regression coefficient) AND variation (e.g. standard deviation) or associated estimates of uncertainty (e.g. confidence intervals)
☐	☒	For null hypothesis testing, the test statistic (e.g. F, t, r) with confidence intervals, effect sizes, degrees of freedom and P value noted<br>*Give P values as exact values whenever suitable.*
☒	☐	For Bayesian analysis, information on the choice of priors and Markov chain Monte Carlo settings
☒	☐	For hierarchical and complex designs, identification of the appropriate level for tests and full reporting of outcomes
☒	☐	Estimates of effect sizes (e.g. Cohen's *d*, Pearson's *r*), indicating how they were calculated

Our web collection on statistics for biologists contains articles on many of the points above.

Software and code

Policy information about availability of computer code

Data collection	IP1 measurements were acquired using PHERAstar FS with the program PHERAstar control Version 4.00 R4.<br>Intracellular Ca2+ release measurements were acquired using Flexstation 3 (Molecular Devices, Sunnyvale, CA, USA) with the program SoftMaxPro 5.4.6.<br>Elisa measurements were acquired using 2103 EnVision Multilabel Plate Reader (Perkin Elmer, Waltham, MA, USA).<br>BRET measurements were acquired using Mithras LB 940 multimode microplate reader (Berthold Technologies, Bad Wildbad, Germany) with the program MikroWin, Version 4.41.<br>Image of gel were collected using an Odyssey infrared scanner (LI-COR Biosciences, Lincoln, NE, USA) at 700nm.
Data analysis	Data were plotted and statistically analyzed using Prism (Versions 7, GraphPad Software).Concentration-responses curve parameters were derived using a four parameters non-linear regression equation. Unless stated otherwise, data shown in the figures represent the mean ± S.E.M. of at least three independent experiments. Statistical differences were determined by GraphPad Prism using one-way ANOVA with a Dunnett's multiple comparison test or two-way ANOVA with Tukey's multiple comparisons test.

For manuscripts utilizing custom algorithms or software that are central to the research but not yet described in published literature, software must be made available to editors and reviewers. We strongly encourage code deposition in a community repository (e.g. GitHub). See the Nature Portfolio guidelines for submitting code & software for further information.

Data

Policy information about availability of data

All manuscripts must include a data availability statement. This statement should provide the following information, where applicable:

- Accession codes, unique identifiers, or web links for publicly available datasets
- A description of any restrictions on data availability
- For clinical datasets or third party data, please ensure that the statement adheres to our policy

Data supporting the findings of this manuscript are available from the corresponding authors upon reasonable request. The source data behind the graphs in the manuscript are shown in Supplementary Data.

Research involving human participants, their data, or biological material

Policy information about studies with human participants or human data. See also policy information about sex, gender (identity/presentation), and sexual orientation and race, ethnicity and racism.

Reporting on sex and gender	N/A
Reporting on race, ethnicity, or other socially relevant groupings	N/A
Population characteristics	N/A
Recruitment	N/A
Ethics oversight	N/A

Note that full information on the approval of the study protocol must also be provided in the manuscript.

Field-specific reporting

Please select the one below that is the best fit for your research. If you are not sure, read the appropriate sections before making your selection.

☒ Life sciences ☐ Behavioural & social sciences ☐ Ecological, evolutionary & environmental sciences

For a reference copy of the document with all sections, see nature.com/documents/nr-reporting-summary-flat.pdf

Life sciences study design

All studies must disclose on these points even when the disclosure is negative.

Sample size	Sample sizes were not predetermined by any statistical metrics, but is consistent with those of other similar reports in the literature for GPCR functional assays. Sample size for each experiments were determined based on standards for experimental cell biology, with a minimum of n = 3 biological independent replicates with sufficient reproducibility. All the presented data are mean ± SEM or representative results for at least three experiments performed independently in triplicate or quadruplicate. Information on the number of replicates and independent experiments that were performed for each measurement is provided in the manuscript.
Data exclusions	No data were systematically excluded. Some individual outliers in the triplicate which was obviously vary from the others, were excluded from the analysis.
Replication	Number of independent experiments and replicates are indicated in the legends to the figures. Experimental findings were reliably reproduced.
Randomization	No randomization was attempted or needed. Randomization was not necessary as the independent variables to be tested were sufficient for the functional interpretation within this study.
Blinding	Blinding was not applicable to this study. All experimental data were acquired using automated equipment and analyzed using computational softwares, eliminating human error and bias.

Reporting for specific materials, systems and methods

We require information from authors about some types of materials, experimental systems and methods used in many studies. Here, indicate whether each material, system or method listed is relevant to your study. If you are not sure if a list item applies to your research, read the appropriate section before selecting a response.

Materials & experimental systems

n/a	Involved in the study	
☐	☒	Antibodies
☐	☒	Eukaryotic cell lines
☒	☐	Palaeontology and archaeology
☒	☐	Animals and other organisms
☒	☐	Clinical data
☒	☐	Dual use research of concern
☒	☐	Plants

Methods

n/a	Involved in the study	
☒	☐	ChIP-seq
☒	☐	Flow cytometry
☒	☐	MRI-based neuroimaging

Antibodies

Antibodies used

Anti-p44/42 ERK Rabbit pAb (9101, 1:3000, Cell Signaling Technology, Shanghai, China)
Anti-phospho-p44/42 ERK Rabbit pAb (9102, 1:3000, Cell Signaling Technology, Shanghai, China)
Anti-Flag Rabbit mAb (KM8002, 1:1000, Sungene Biotech, Tianjin Province, China)
Anti-α-tubulin Mouse pAb (KM9007 1:3000, Sungene Biotech, Tianjin Province, China)
Anti-mouse IgG DyLight 800 4X PEG conjugated secondary antibodies (#5257, 1:20000, Cell Signaling Technology,)
Anti-rabbit IgG DyLight 800 4X PEG conjugated secondary antibodies (#5151, 1:20000, Cell Signaling Technology, Shanghai, China)
Anti-Flag Rabbit mAb coupled with horseradish peroxidase(F1804, 1:20000, Sigma-Aldrich,Shanghai, China)
Anti-HA Rabbit mAb coupled with horseradish peroxidase(3F10, 1:20000, Roche, Indianapolis, USA)

Validation

Commercial antibodies used in this study are widely used and have been validated by the respective manufacturer.
Anti-p44/42 ERK Cell Signaling Technology Cat#9101: 8215 citations reported on manufacturer's website (https://www.cellsignal.cn/products/primary-antibodies/phospho-p44-42-mapk-erk1-2-thr202-tyr204-antibody/9101)
Anti-phospho-p44/42 ERK Cell Signaling Technology Cat#9102: 7376 citations reported on manufacturer's website (https://www.cellsignal.cn/products/primary-antibodies/p44-42-mapk-erk1-2-antibody/9102)
Anti-Flag Sungene Biotech Cat#KM8002: Validated by company (http://www.sungenebiotech.com/index.php?m=Product&a=product_xq&catid=2&proid=54&prid=293&pid=695&id=1503)
Anti-α-tubulin Sungene Biotech Cat#KM9007: Validated by company (http://www.sungenebiotech.com/index.php?m=Product&a=product_xq&catid=2&proid=53&prid=291&pid=723&id=1566)
Anti-mouse IgG DyLight 800 4X PEG conjugated secondary antibodies Cell Signaling Technology Cat#5257: 129 citations reported on manufacturer's website (https://www.cellsignal.cn/products/secondary-antibodies/anti-mouse-igg-h-l-dylight-8482-800-4x-peg-conjugate/5257)
Anti-rabbit IgG DyLight 800 4X PEG conjugated secondary antibodies Cell Signaling Technology Cat#5151: 244 citations reported on manufacturer's website (https://www.cellsignal.cn/products/secondary-antibodies/anti-rabbit-igg-h-l-dylight-800-4x-peg-conjugate/5151)
Anti-Flag Rabbit mAb coupled with horseradish peroxidase Cat#F1804: Validated by company(https://www.sigmaaldrich.cn/CN/zh/product/sigma/f1804#product-documentation)
Anti-HA Rabbit mAb coupled with horseradish peroxidase Cat#3F10: Validated by company(https://www.sigmaaldrich.cn/CN/zh/product/roche/12158167001)

Eukaryotic cell lines

Policy information about cell lines and Sex and Gender in Research

Cell line source(s)	HEK293 cells (ATCC, CRL-1573, lot: 3449904) were used.
Authentication	HEK293 cell line was obtained from ATTC and used without further authentication.
Mycoplasma contamination	HEK293 cells were tested monthly in laboratory and no mycoplasma contamination was found, which was described in the manuscript.
Commonly misidentified lines (See ICLAC register)	No commonly misidentified cell lines were used in this study.

Plants

Seed stocks	N/A
Novel plant genotypes	N/A
Authentication	N/A
